# Recombinational exchange of M-fibril and T-pilus genes generates extensive cell surface diversity in the global group A *Streptococcus* population

**DOI:** 10.1128/mbio.00693-24

**Published:** 2024-04-09

**Authors:** Debra E. Bessen, Bernard W. Beall, Andrew Hayes, Weihua Huang, Jeanne M. DiChiara, Srinivasan Velusamy, Hervé Tettelin, Keith A. Jolley, John T. Fallon, Sopio Chochua, Mosaed S. A. Alobaidallah, Charlie Higgs, Timothy C. Barnett, John T. Steemson, Thomas Proft, Mark R. Davies

**Affiliations:** 1 Department of Pathology, Microbiology, and Immunology, New York Medical College, Valhalla, New York, USA; 2 Respiratory Disease Branch, National Center for Immunizations and Respiratory Diseases, Centers for Disease Control and Prevention (CDC), Atlanta, Georgia, USA; 3 Eagle Global Scientific, LLC, Atlanta, Georgia, USA; 4 Department of Microbiology and Immunology, The University of Melbourne at the Peter Doherty Institute for Infection and Immunity, Melbourne, Australia; 5 Department of Pathology, Brody School of Medicine, Eastern Carolina University, Greenville, North Carolina, USA; 6 Department of Microbiology and Immunology, Institute for Genome Sciences, University of Maryland School of Medicine, Baltimore, Maryland, USA; 7 Department of Biology, University of Oxford, Oxford, United Kingdom; 8 The Marshall Centre for Infectious Diseases Research and Training, School of Biomedical Sciences, University of Western Australia, Nedlands, Australia; 9 Wesfarmers Centre of Vaccines and Infectious Diseases, Telethon Kids Institute, University of Western Australia, Nedlands, Australia; 10 School of Biological Sciences, The University of Auckland, Auckland, New Zealand; 11 School of Medical and Health Sciences, The University of Auckland, Auckland, New Zealand; University of Colorado Anschutz Medical Campus, Aurora, Colorado, USA; ^1^Washington University School of Medicine, St. Louis, Missouri; University of Maryland, College Park, Maryland, USA; Imperial College London, London, England

**Keywords:** group A streptococcus, pili, genotyping, cell surface proteins, population biology, molecular epidemiology

## Abstract

Among genes present in all group A streptococci (GAS), those encoding M-fibril and T-pilus proteins display the highest levels of sequence diversity, giving rise to the two primary serological typing schemes historically used to define strain. A new genotyping scheme for the pilin adhesin and backbone genes is developed and, when combined with *emm* typing, provides an account of the global GAS strain population. Cluster analysis based on nucleotide sequence similarity assigns most T-serotypes to discrete pilin backbone sequence clusters, yet the established T-types correspond to only half the clusters. The major pilin adhesin and backbone sequence clusters yield 98 unique combinations, defined as “pilin types.” Numerous horizontal transfer events that involve pilin or *emm* genes generate extensive antigenic and functional diversity on the bacterial cell surface and lead to the emergence of new strains. Inferred pilin genotypes applied to a meta-analysis of global population-based collections of pharyngitis and impetigo isolates reveal highly significant associations between pilin genotypes and GAS infection at distinct ecological niches, consistent with a role for pilin gene products in adaptive evolution. Integration of *emm* and pilin typing into open-access online tools (pubmlst.org) ensures broad utility for end-users wanting to determine the architecture of M-fibril and T-pilus genes from genome assemblies.

**IMPORTANCE:**

Precision in defining the variant forms of infectious agents is critical to understanding their population biology and the epidemiology of associated diseases. Group A *Streptococcus* (GAS) is a global pathogen that causes a wide range of diseases and displays a highly diverse cell surface due to the antigenic heterogeneity of M-fibril and T-pilus proteins which also act as virulence factors of varied functions. *emm* genotyping is well-established and highly utilized, but there is no counterpart for pilin genes. A global GAS collection provides the basis for a comprehensive pilin typing scheme, and online tools for determining *emm* and pilin genotypes are developed. Application of these tools reveals the expansion of structural-functional diversity among GAS via horizontal gene transfer, as evidenced by unique combinations of surface protein genes. Pilin and *emm* genotype correlations with superficial throat vs skin infection provide new insights on the molecular determinants underlying key ecological and epidemiological trends.

## INTRODUCTION

Group A *Streptococcus* (GAS) is a strictly human pathogen causing ~750 million infections per year (1). Most GAS infections occur at the superficial epithelia of the throat or skin, resulting in pharyngitis or impetigo, respectively. During infection, GAS reproduces in its host to generate high numbers of progeny, and it is the epithelia of the throat and skin from which many/most transmissions to new hosts are launched. The primary modes of transmission differ for organisms causing pharyngitis (respiratory) vs impetigo (direct contact), leading to different sets of risk factors (2, 3). These in turn impart distinct epidemiological trends: A higher prevalence of pharyngitis in temperate regions (and winter-early spring) and a higher prevalence of impetigo in tropical regions (and summer elsewhere). The multiple spatial and temporal distances that separate GAS causing pharyngitis vs impetigo have the potential to impact horizontal gene transfer (HGT) and homologous recombination (4). Thus, the population genetic structure of the species is shaped by a series of feedback loops operating on several different orders of scale (3).

GAS organisms are rich in the molecular diversity of their cell surface proteins (5). Historically, two main serological typing schemes were used to characterize GAS “strains.” M-typing is based on the short M protein surface fibril, and T-typing is based on trypsin-resistant heteropolymers forming elongated pili (Fig. S1). In addition to high-sequence diversity, M proteins and pili are key virulence factors and targets of host protective immunity (6–11). A third serotyping scheme that was often used to help resolve a subset of GAS organisms is based on the SOF (serum opacity factor) protein (12).

There is a widely adopted genotyping scheme for M protein (i.e., *emm* type) (13–16). The concept of “strain” based strictly on *emm* type overlooks the extremely high level of HGT that occurs among GAS organisms (4, 17–24), with numerous *emm* types emerging on distant genetic backgrounds (24–26). There is no readily accessible genotyping scheme for the pilin genes that give rise to T serotype. Many organisms have a tip adhesin and multimers of the backbone subunit comprising the “shaft” (7, 27, 28) (Fig. S1). Purified recombinant polypeptides corresponding to adhesin and backbone subunits are bound by T-typing serum, and some/many are bound by multiple T-typing sera (29, 30). Likewise, numerous GAS organisms are agglutinated by multiple T-typing sera (5, 31). The relationships between T-typing serum and adhesin and backbone subunits are overlapping and complex.

The goals of this study are to develop a pilin genotyping scheme, define the unique pairings of *emm* and pilin types and their past horizontal exchange within a global collection of GAS, and evaluate the relationships between *emm* and pilin genotypes for GAS isolates from the two primary tissue sites for infection. This effort aims to deepen understanding of the population biology of this important pathogen and provide an expanded toolset for future epidemiological studies.

## RESULTS AND DISCUSSION

### Unifying nomenclature of pilin antigens from a diverse GAS genome database

**Fig 1:**
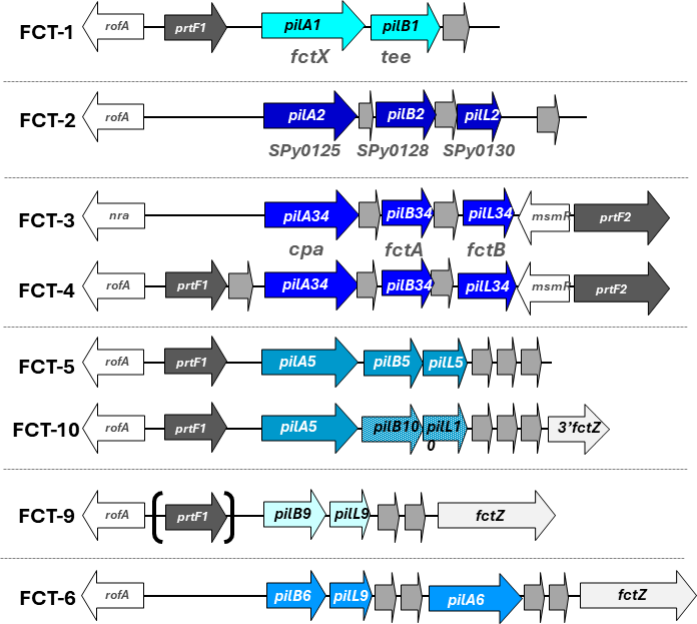
FCT-region forms of GAS. Nomenclature is proposed for pilin subunit adhesin (*pilA*), backbone (*pilB*), and linker (*pilL*) loci (arrows), for each of the eight FCT-region forms present in genomes of 628 GAS isolates. Horizontal dotted lines separate the FCT-region forms corresponding to the six major lineages of pilin loci (shades of blue). Other loci encode transcriptional regulators (arrows, no fill), fibronectin-binding proteins (dark gray), pilus biosynthesis enzymes (medium gray), and a putative LPxTG-linked surface protein (designated *fctZ*; light gray) sharing weak homology with the pilin-like homolog of Spa from *Corynebacterium diphtheriae*. Note that FCT-11 described in reference (34) is renamed FCT-10 based on its similarity to that described in reference (35). The FCT-9 region has either a full-length or partial *prtF1* locus. FCT-regions range from ~11 to 16 kb (33); FCT-7 and FCT-8, as originally defined by PCR-based mapping (32), have not been confirmed via WGS. The alternative nomenclature of Table S2 is also indicated (light gray font).

A highly diverse sample set of 628 organisms was selected from a larger collection of >3,000 whole-genome sequences (WGS) based on unique combinations of *emm* type, multi-locus sequence type (MLST), geographic region, and/or date of isolation (Table S1). Included are representatives of 169 *emm* types recovered from >35 countries, spanning >100 years. All genomes harbor one FCT-region (32, 33) (Table S1), assigned to eight forms based on overall genetic architecture (Fig. 1). To aid in the systematic profiling of pilin genes, a unifying nomenclature is proposed for adhesin (*pilA*), backbone (*pilB*), and linker (*pilL*) subunit loci (Table S2). All genomes contain *pilB*, with *pilA* and *pilL* absent from FCT-9 and FCT-1, respectively (Fig. 1). Ten new gap-free reference genomes representing novel GAS genotypes are also reported (Table 1).

**TABLE 1:** Gap-free (closed) WGSs for 10 GAS strains^*a*^

Strain name	Size (bp)	*emm* type	*emm* subtype	*emm* pattern	FCT-region form	ST	Origin	Year	Tissue	Disease	Source
6745–99	1,842,075	4	4.0	E	FCT-5	105	Brazil	1999	Impetigo	impetigo	CDC
6702–99	1,814,755	14	14.4	A–C	FCT-4	118	USA	1999	Sterile site	iGAS	CDC
11RS100	1,841,219	26	26.0	A–C	FCT-5	158	USA	1942	URT	carriage	Lancefield
A946	1,821,159	52	52.1	D	FCT-3	43	Trinidad	1971	Impetigo	impetigo	Lancefield
AW-534	1,793,445	52	52.2	D	FCT-3	270	Ethiopia	1990	Impetigo	impetigo	Tewodros
CT95-157	1,741,389	78	78.3	E	FCT-4	253	USA	1995	Sterile site	iGAS	CT DOH
D641	1,749,382	101	101.1	D	FCT-3	11	Trinidad	1972	Impetigo	impetigo	Lancefield
SS1366	1,788,060	115	115.0	D	FCT-1	135	USA	1995	Sterile site	iGAS	CDC
2907–97	1,846,043	115	115.0	D	FCT-3	123	Brazil	1997	URT	n.d.	CDC
SS1445	1,805,137	218	218.2	A–C	FCT-4	145	Brazil	1997	URT	n.d.	CDC

^*a*^ ST, sequence type (based on MLST); iGAS, invasive GAS; URT, upper respiratory tract.

### Cluster analysis of pilin adhesin and backbone genes and their products

Collectively, 257 pilin adhesin and 219 pilin backbone alleles corresponding to full-length genes were identified among the 628 genome sequences (Table 2). Key properties of the *pilA* and *pilB* alleles and their products are described (Tables S3 and S4). Cluster analysis was performed for the 257 adhesin and 219 backbone alleles and their translated products. Using a low similarity threshold cutoff of 50% amino acid identity (aa50) over 90% coverage, six PilA clusters and 11 PilB clusters are defined. The 11 PilB clusters closely align with sequence length and the number of immunoglobulin (Ig)-like domains within each PilB subunit (Table S4) (36, 37).

**TABLE 2:** Distribution of *pilA* and *pilB* alleles among ancestral lineages defined by low threshold (aa50) clustering

FCT-region form	No. of isolates	No. of *pilA* alleles^*a*^	No. of PilA clusters (aa50)^*b*^	No. of *pilB* alleles	No. of PilB clusters (aa50)
FCT-1	54	30	1	24	1
FCT-2	14	5	1^*c*^	2	1
FCT-3 & 4	424	186	3	137	1
FCT-5 & 10	57	35	1	26^*d*^	3
FCT-6	6	1	1	1	1^*e*^
FCT-9	73	0	n/a	29	5
Total	628	257	6	219	11

^*a*^ Allele is defined by a single SNP (single nucleotide polymorphism) difference.

^*b*^ The cd-hit settings for aa50 clusters are aa50-s90-n4-g1.

^*c*^ The FCT-2 PilA lineage overlaps with a PilA aa50 cluster from FCT-34 isolates (Fig. 2A ).

^*d*^ For the structurally related FCT-5 and FCT-10 regions, there are 20 *pilB*5 alleles and six *pilB*10 alleles, assigned to one and two aa50 clusters, respectively.

^*e*^ The FCT-6 PilB lineage overlaps with a PilB aa50 cluster from FCT-9 isolates (Fig. 2B ).

**Fig 2:**
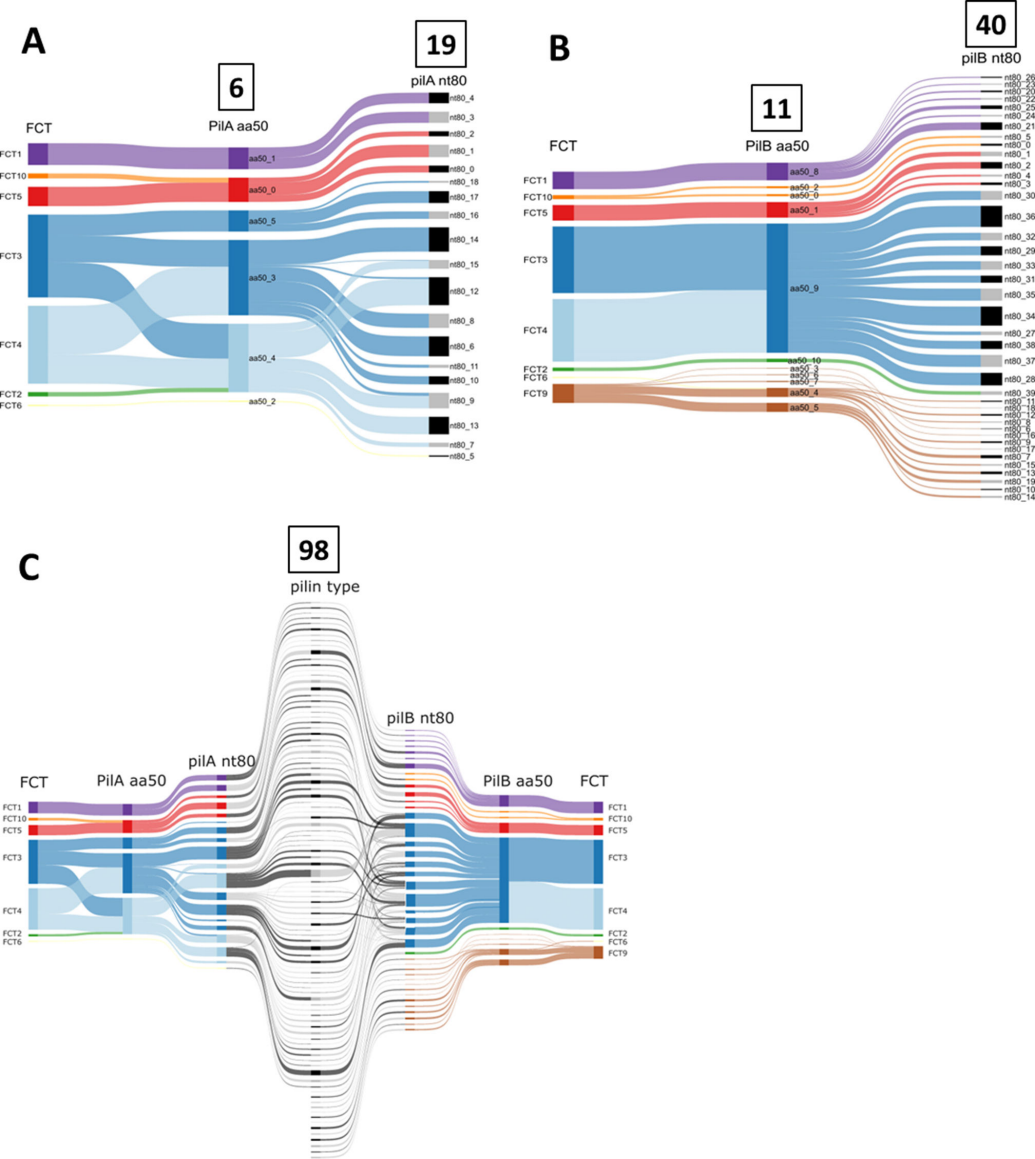
Sankey diagram for visualizing pilin sequence clusters. FCT-region assignment (colors) was determined for 628 genomes (FCT-9 lacks the *pilA* locus). (**A**) Clustering of 257 pilin adhesin sequences at two thresholds: amino acid similarity at 50% over 90% sequence length (PilA aa50) and a *pilA* gene homology at 80% sequence identity and length (*pilA* nt80). (**B**) Clustering of 219 pilin backbone sequences, as described for pilin adhesin sequences. The number of clusters at each threshold is indicated (boxed). (**C**) Merged Sankey plots for pilin adhesin and backbone sequences, illustrating intra-FCT-region crossover events and unique pilin types.

The PilA and PilB cluster assignments display close correspondence to FCT-region forms, indicating discrete ancestral lineages (Fig. 2A and B). FCT-3 and FCT-4 (FCT-34) isolates have three distinct PilA lineages, wherein one lineage overlaps with PilA from FCT-2. However, PilB sequences of FCT-34 isolates consist of a single aa50 lineage that is distinct from the PilB lineage of FCT-2 organisms. FCT-9 isolates lack the *pilA* locus but harbor five highly divergent aa50 clusters for PilB, one of which overlaps with PilB from FCT-6 organisms. All FCT-5 and FCT-10 isolates share a PilA lineage but differ in their PilB aa50 clusters.

For a more refined analysis of the genetic relatedness among pilin alleles, sequential clustering was applied to nucleotide (nt) sequences of *pilA* and *pilB* alleles over a broad range of percent sequence identity threshold (Fig. S2A and B). As the nucleotide sequence cutoff is lowered from 95% (nt95) to 80% (nt80), there is a stepwise decrease in the number of clusters for *pilA* and *pilB* loci, with nt80 most closely positioned at an inflection point. The 19 *pilA* and 40 *pilB* nt80 clusters are highly congruent with FCT-region forms (Fig. 2A and B). *pilL* alleles were determined for many genomes (Table S1) but did not undergo cluster analysis due to high levels of sequence homogeneity within an FCT-region form (data not shown).

Findings on pilin cluster assignments closely parallel phylogenetic analysis. For *pilB*34 alleles, nt80 clusters for 135 of the 137 alleles closely align with well-supported branches of a phylogenetic tree (Fig. S3A). BLASTN analysis of one outlier allele (*pilB*34_136) reveals the highest homology to a *Streptococcus dysgalactiae* subspecies *equisimilis* strain (DY107; CP082206.1) FCT-region-like gene, suggestive of interspecies exchange. The second allele lacking strong branch support (*pilB*34_50; cluster nt80_34) shows evidence of intragenic recombination with cluster nt80_32 alleles at its 5’ end (data not shown). The high concordance between *pilB*34 cluster and phylogenetic lineage permits newly discovered alleles to be readily assigned a cluster group (red dots, Fig. S3A), providing a practical approach for future studies.

### Relationships between pilin genotypes and T-serotype reference strains

Historically, T-protein agglutination was often the first step in categorizing GAS isolates as discrete “strains” (38). T-typing serum is generated by immunizing rabbits with whole bacterial cells, followed by the removal of cross-reactive antibody via absorption to GAS of other T-types. Whole bacterial cells are pre-digested with trypsin prior to serum agglutination, and therefore, antigenic epitopes targeted by T-typing may differ from those naturally exposed on the bacterial cell surface during human infection.

To examine relationships between the prototypical T-serotypes and pilin genotypes, the 21 reference T-typing strains used by the Centers for Disease Control and Prevention (USA) underwent WGS. Cluster analysis findings for *pilB* genes of the 21 reference strains are shown (Table 3). The 80% nt id cutoff (nt80) yields 18 *pilB* clusters; however, raising the threshold to nt92 or nt95 fails to increase the number of clusters for the T-typing strains. Matches to the partial *pilB* allele sequences used in the CDC genomics pipeline (39) and TeeVax vaccine (37, 40) are provided for interest (Tables S5 and S6).

**TABLE 3:** Pilin genotypes of T-serotype reference strains

T-type	Typing strain^*a*^	*emm* type^*b*^	FCT-region	*pilB* allele	PilB aa50^*c*^	PilB aa70	*pilB* nt80	*pilB* nt92	*pilA* nt80
T23	SS580	23	FCT-1	*pilB*1_4	**aa50_8**	aa70_14	nt80_21	nt92_27	
T6	SS625	6	FCT-1	*pilB*1_6	**aa50_8**	aa70_15	nt80_25	nt92_28	
T4	SS691	4	FCT-5	*pilB*5_4	aa50_1	aa70_1	nt80_1	nt92_1	
T22	SS579	22	FCT-10	*pilB*10_3	aa50_0	aa70_0	nt80_0	nt92_0	
T1	SS566	166	FCT-2	*pilB*2_1	aa50_10	aa70_26	nt80_39	nt92_50	
T11	SS574	11	FCT-34	*pilB*34_12	**aa50_9**	aa70_17	nt80_27	nt92_30	
T-B^*d*^	SS585	68	FCT-34	*pilB*34_34	**aa50_9**	aa70_19	nt80_30	nt92_33	
T9	SS573	9	FCT-34	*pilB*34_47	**aa50_9**	aa70_21	nt80_32	nt92_35	
T44	SS589	44	FCT-34	*pilB*34_107	**aa50_9**	**aa70_22**	**nt80_33**	**nt92_38**	nt80_8
T5	SS570	5	FCT-34	*pilB*34_36	**aa50_9**	**aa70_22**	**nt80_33**	**nt92_38**	nt80_18
T27	SS582	Null	FCT-34	*pilB*34_67	**aa50_9**	**aa70_22**	**nt80_33**	**nt92_38**	nt80_11
T12	SS593	12	FCT-34	*pilB*34_15	**aa50_9**	**aa70_22**	nt80_38	nt92_48	
T13	SS576	13	FCT-34	*pilB*34_53	**aa50_9**	**aa70_23**	nt80_29	nt92_36	
T28	SS583	28	FCT-34	*pilB*34_83	**aa50_9**	**aa70_23**	nt80_34	nt92_42	
T3	SS568	3	FCT-34	*pilB*34_79	**aa50_9**	aa70_24	nt80_35	nt92_46	
T14	SS577	51	FCT-34	*pilB*34_23	**aa50_9**	**aa70_25**	**nt80_36**	**nt92_44**	nt80_11
T18	SS578	18	FCT-34	*pilB*34_64	**aa50_9**	**aa70_25**	**nt80_36**	**nt92_44**	nt80_17
T2	SS567	Null	FCT-6	*pilB*6_1	**aa50_4**	aa70_5	nt80_11	nt92_13	
T-Imp19	SS586	92	FCT-9	*pilB*9_3	**aa50_4**	aa70_7	nt80_12	nt92_14	
T8	SS572	8	FCT-9	*pilB*9_13	**aa50_5**	aa70_12	nt80_19	nt92_21	
T25	SS581	25	FCT-9	*pilB*9_2	**aa50_5**	aa70_9	nt80_14	nt92_16	

^*a*^ All 21 CDC T-typing reference strains underwent WGS from CDC stocks; many of these strains are shared by multiple labs, and wherever NCTC accession numbers are reported instead of CDC stocks (Table S1), there were no sequence discrepancies noted.

^*b*^ As with M-serotyping strains, many T-typing reference strains were subject to extensive lab manipulation and passage to maximize expression of antigenic targets; this procedure may explain the loss of *emm* in several T-typing strains (i.e., T1, T2, and T27).

^*c*^ Sequence clusters shared by >1 of the *pilB* alleles listed (e.g., aa50_9, aa70_22, etc.) are **bolded** according to each cluster threshold.

^*d*^ Known as T-B3264 and abbreviated here.

In the global collection of 628 GAS genomes, 40 *pilB* nt80 clusters are identified (Fig. S2B). Thus, the 21 T-typing strains fail to account for roughly half of the *pilB* nt80 clusters. Six additional T-types are omitted from more recent T-serotyping practices (38); their corresponding *pilB* clusters remain unknown. The *pilB*1 and *pilB*9 loci yield 7 and 13 nt80 clusters, respectively (Fig. S2B); however, *pilB*1 and *pilB*9 correspond to only two or three T-types, respectively (Table 3). Thus, most *pilB*1 and *pilB*9 nt80 clusters (15 out of 20) lack a corresponding T-typing strain.

For further analysis of *pilB*9 alleles, SplitsTree graphs are constructed, and deep branching is observed for each nt80 cluster (Fig. S4A). When non-*pilB*9 genes are added (Fig. S4B), the position of the *pilB*6_1 allele (from the T2 typing strain) confirms the ancestral overlap of the FCT-9 and FCT-6 forms. The close correspondence of *pilB*-like gene taxa from other streptococcal species (green) illustrates that intragenic recombination involving *pilB*9 alleles may be coupled to interspecies gene exchange.

**Fig 3:**
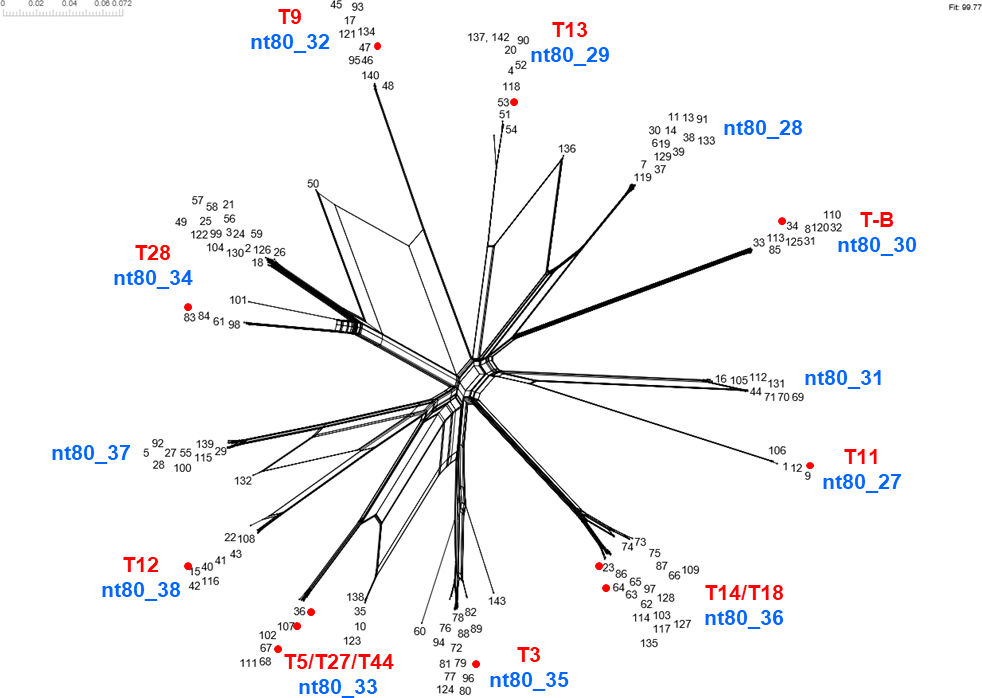
Neighbor-net analysis of *pilB*34 alleles. The 137 *pilB*34 alleles were aligned by MUSCLE and underwent neighbor-net analysis using SplitsTree v5. Alleles corresponding to the 12 T-serotype reference strains harboring a *pilB*34 allele are indicated (red font and red dots). Also marked are the positions of the 12 nt80 clusters identified by cd-hit (blue).

Of the 11 *pilB* (backbone) aa50 ancestral lineage-like clusters (Table 2), alleles of just one ancestral group are shared by all organisms having FCT-3 or FCT-4 region forms. A SplitsTree graph provides evidence for intragenic recombination involving *pilB*34 alleles but also shows clear partitioning of nt80 clusters (Fig. 3), as observed in the phylogenetic tree (Fig. S2A). The 137 *pilB*34 alleles generate 12 nt80 cluster groups and correspond to 12 T-typing sera (Table 3); however, those relationships are not one-to-one. Two of the *pilB*34 nt80 clusters correspond to >1 T-type reference strain (Fig. 3; Table 3, bolded). *pilB*34 backbone alleles of the T5, T27, and T44 reference strains display 99.2% nt and 99.4% aa identities, but importantly, each has *pilA*34 adhesin alleles of different nt80 clusters (Table 3). Similarly, the *pilB*34 alleles of the T14 and T18 reference strains share a nt92 cluster and display 99.2% nt and 98.6% aa identity yet have distinct *pilA*34 nt80 clusters. Thus, the PilB sequence does not appear to distinguish among these T-serotypes. Alignment of the predicted amino acid sequences of the mature PilB34 polypeptides of the 12 T-typing strains shows that few residue positions are identical, with no conserved segments exceeding four residues in length (Fig S3B).

The disparities in correspondence between T-serotype and *pilB* genotype, combined with the ease and precision of nucleotide sequence determination, provide the impetus for developing a pilin genotyping scheme that can distinguish among the many variants of this important cell surface antigen and virulence factor. Based on methodical analysis of sequence clusters, the nt80 threshold cutoff provides a good balance for distinguishing (nearly all) T-serotypes, while also minimizing the number of sequence clusters that fail to match an established T-serotype.

### A practical genotype-based definition for “pilin type”

The proposed definition for GAS pilin type is the unique combination of nt80 clusters for *pilA* (adhesin) and *pilB* (backbone) full-length alleles of an organism. The 628 global isolates of the data set yield 98 distinct pilin types (Table 4; Fig. 2C).

**TABLE 4:** Pilin type defined as a unique combination of *pilA* and *pilB* nt80 clusters

FCT-region form	No. of *pilA* nt80 clusters	No. of *pilB* nt80 clusters	No. of unique *pilA-pilB* nt80 cluster combinations (pilin types)
FCT-1	2	7	10
FCT-2	1	1	1
FCT-34	12	12	64
FCT-5	2	4	7
FCT-10	1	2	2
FCT-6	1	1	1
FCT-9	0	13	13
Totals	19	40	98

A unique pilin type is less stringently defined than *emm* type, which is based on the first 30 codons encoding the mature M protein and a 92% nucleotide sequence identity threshold allowing for some gaps (Fig. S1) (13). M proteins form an extended coiled-coil α-helix with M-type-specific epitopes localized to the non-helical fibril tips (41–43). In contrast, pilin subunits have surface-exposed epitopes distributed throughout the mature polypeptide length (Fig. S3B) (36). The 80% sequence similarity threshold for pilin nt80 clusters is lower than the threshold for *emm* type and provides a conservative measure for distinguishing structurally and antigenically distinct pili.

Conceptually, our approach parallels a preliminary pilin genotyping plan that included loci for (up to) all three pilin subunits (29) but was never widely adopted. The inclusion of both *pilA* (adhesin) and *pilB* (backbone) in a pilin genotyping scheme is justified by the cell surface exposure of their gene products and binding by T-typing serum (29, 30), coupled with high levels of sequence diversity. The open-access databases and tools provided through the well-established and widely used www.pubmlst.org website (44) make the newly proposed pilin genotyping scheme readily available to all users and complement the addition of *emm* typing databases (45).

To further simplify the newly proposed pilin genotyping scheme, the 98 unique combinations of *pilA* and *pilB* nt80 clusters are assigned numerical pilin types, using the format pil001, pil002, pil003, etc. (Table S1). For many pilin types, the numbers match a dominant T-serotype and/or *emm* type. For example, an M1T1 isolate has the genotype emm1/pil001. In other instances, the numerical pilin type is randomly assigned, with no relationship to the numerical M- or T-type. The objective is a nomenclature that is easy to use and may often convey genotype information which in turn might have biological relevance. Pilin type can be used to concisely describe GAS strains of high interest, such as the emm89-pil089 strain that recently rose in dominance and gradually replaced its progenitor emm89-pil011 strain differing in only a single MLST housekeeping allele (3, 46–48).

### The strain concept and unique combinations of *emm* and pilin types

GAS “strains” are often denoted by *emm* type or M-serotype, which is an oversimplification because of the rich evolutionary history of extensive HGT of *emm* to recipient cells having distant genetic backgrounds (24, 25). The FCT- and *emm*-regions map ~280 kb apart on the ~1.85 Mb chromosome, ~equidistant from the origin of replication.

Like the FCT-region, the *emm* chromosomal region is structurally complex and harbors several key virulence genes (Fig. S5). Collectively, FCT- and *emm*-region gene products bind numerous plasma and extracellular matrix proteins of the human host and supply the bulk of antigenic diversity present on the GAS cell surface (34, 43, 49, 50). Serotype-specific epitopes of both the M-fibrils and T-pilus are targets of protective immunity and provide the basis for several GAS vaccines under development (6, 40, 51).

**Fig 4:**
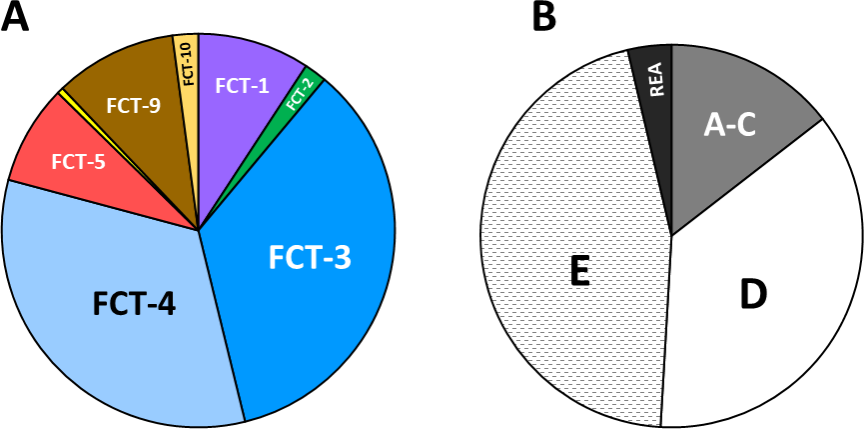
FCT-region forms and *emm* pattern groups for the set of unique *emm*-pilin type combinations. Fractional distribution of 379 GAS organisms having unique combinations of *emm* and pilin type, according to FCT-region form (**A**) and *emm* pattern grouping (**B**); REA, rearranged; yellow (unlabeled), FCT-6.

Among the 628 GAS genomes—chosen for study based on their known genetic and geographic diversity—are 379 unique combinations of *emm* and pilin type (Table S1). The set of 379 genetically diverse GAS is dominated by FCT-3 and FCT-4 region genotypes (68%; Fig. 4A) and by *emm* pattern groupings D and E (82%; Fig. 4B).

**Fig 5:**
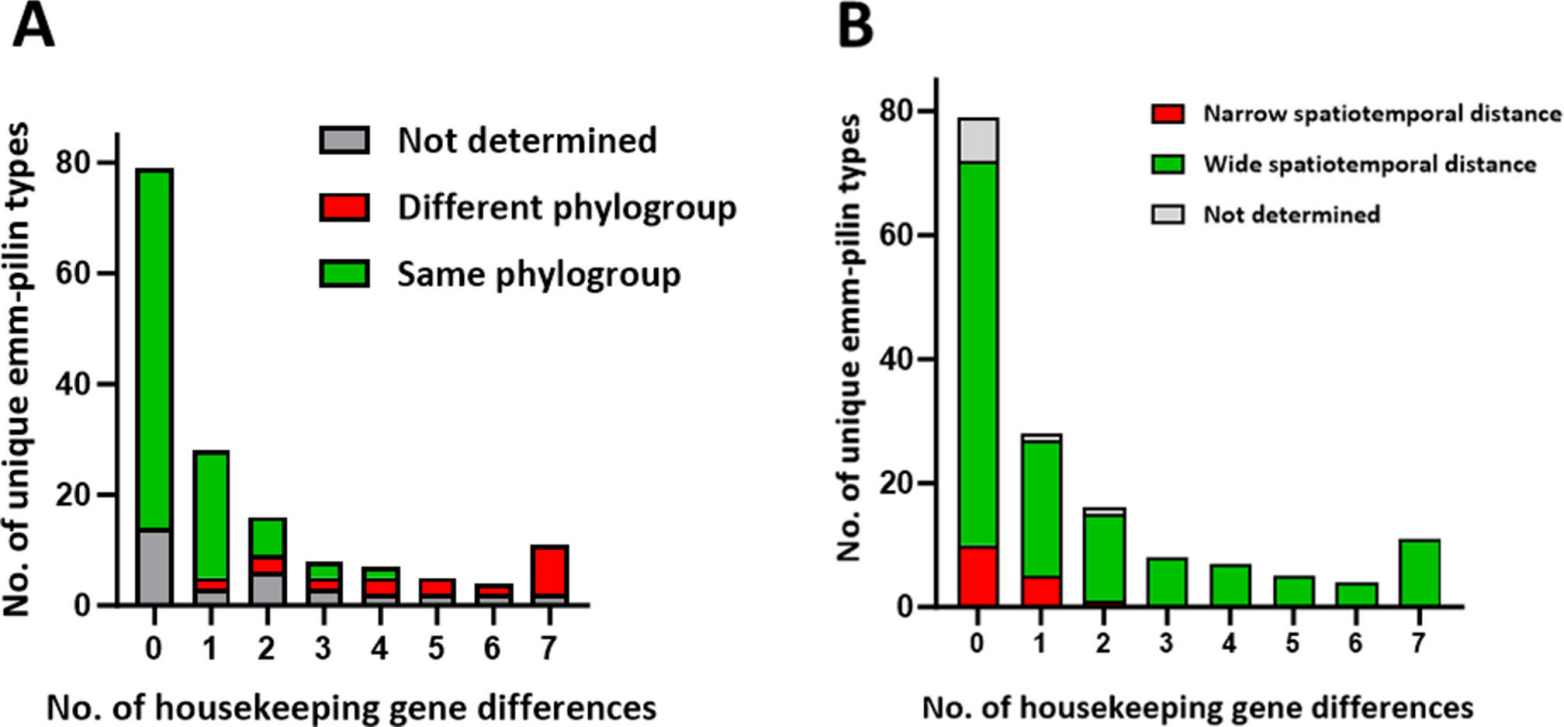
Genetic distances among 158 unique *emm*-pilin types. Each unique *emm*-pilin type having multiple isolates in the data set is analyzed for the maximum number of core housekeeping gene differences among those isolates (x-axis). (**A**) Distribution of same vs different PopPUNK phylogroups (whole-genome clusters). (**B**) Distribution of wide vs narrow spatiotemporal distances. A wide spatiotemporal distance is defined as the recovery of multiple isolates from different countries and/or >2 years apart, based on Table S1 data.

Of the 379 unique *emm*-pilin type combinations, 158 *emm*-pilin types are represented by multiple GAS isolates in the data set of 628 genomes. For these genotypes, the relationships between *emm*-pilin type and other chromosomal genes are assessed (Fig. 5A). MLST for GAS is based on seven housekeeping loci (52); isolates for 78% of the subset of 158 unique *emm*-pilin types share the same clonal complex (i.e., 0, one or two allele differences; Fig. 5A, bar height). For a broader genome-wide assessment, PopPUNK is used to identify evolutionarily related lineages (i.e., whole-genome clusters) by cluster analysis of core and accessory gene sequences (24, 53). Data show strong concordance between whole-genome clusters and MLST-based sequence types (STs) and like MLST, all isolates of most *emm*-pilin types examined (81%) belong to a single whole-genome cluster (Fig. 5A, green). Shared genetic backgrounds signify recent common ancestors and fit the concept of “strain.”

For 20 (12%) of the unique 158 *emm*-pilin type combinations with multiple isolates, there are >5 allele differences in MLST housekeeping genes (Fig. 5A, bar height); for those organisms also analyzed via PopPUNK, all have distinct whole-genome clusters (Fig. 5A, red). Multiple occurrences of the emergence of the same *emm*-pilin type combination on distant genetic backgrounds are a likely mechanism; however, rapid and extensive genetic change in other core genes is difficult to rule out.

The country and year of recovery are known for most organisms of the 158 unique *emm*-pilin types with multiple isolates in the data set (Table S1), permitting analysis of spatiotemporal distances. Of the 158 *emm*-pilin types, 89% correspond to isolates recovered from different countries and/or >2 years apart (Fig. 5B, red); 87% had isolates from different continents and/or >5 years apart (data not shown). Thus, most organisms sharing an *emm*-pilin type do not originate from the same localized outbreak but rather, persist over time or migrate over great distances. In addition, the data align well with the sampling strategy, which sought unique combinations of *emm* and distant ST, coupled with geographic diversity and long timeframes. The findings underscore the stability of *emm*-pilin type-defined strains across extended time and distant geography.

The strain concept for GAS has utility by defining closely related organisms that often share key clinico-epidemiologic properties and are similarly impacted by immune responses mediated via the human host population. Concordance between *emm*-pilin type combination and ST or whole-genome cluster is high but imperfect. The *emm*-pilin type combination better approximates a working definition for “strain” than does *emm* type alone; however, the addition of a third genotype that incorporates core gene sequences can provide a critical layer of refinement (24, 26, 52, 54).

### Recombinational exchanges involving *emm* and pilin types

**Fig 6:**
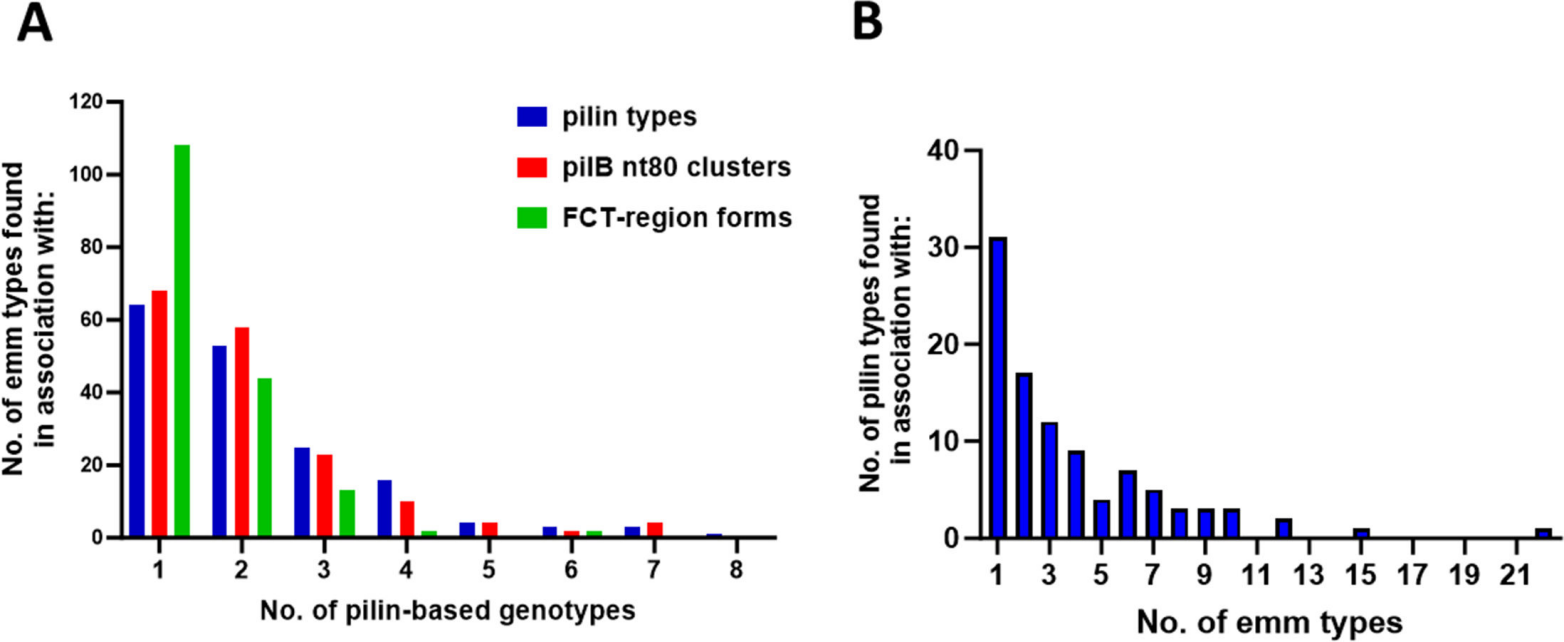
Associations between *emm* and pilin genotypes. (**A**) The number of *emm* types found in association with three pilin genotypes defined over a wide range of resolution (pilin types, blue; *pilB* nt80 clusters, red; FCT-region forms, green). (**B**) The number of pilin types found in association with various numbers of *emm* types. The four *emm*-null organisms are excluded from all calculations.

GAS organisms with novel cell surface properties appear to emerge following HGT of either *emm* or pilin genes (31). En bloc replacement of the FCT-region form or change in *emm* pattern structure can potentially result in a profound shift in the virulence factor repertoire. In Fig. 6, unique mixtures of *emm* and pilin genotypes are evaluated over multiple levels of genetic resolution.

Among the 379 unique *emm*-pilin type combinations, 105 *emm* types (62.1%) are paired with >1 pilin type (Fig. 6A, blue; Table S7 data). Only slightly fewer *emm* types (*N* = 101, or 59.8%) are paired with >1 *pilB* nt80 cluster. The inclusion of *pilA* nt80 clusters in the pilin type definition does not significantly alter the number or distribution of *emm*-pilin genotypes (*P* = 0.87, χ^2^ = 3.16, 7 degrees of freedom; Fig. 6A, blue vs red).

A relatively high number of *emm* types (*N* = 26 or 15.4%) are paired with >4 pilin types. Associations with multiple FCT-region forms are found for 60 *emm* types (35.5%; Fig. 6A, green), indicative of high plasticity for many *emm*-pilin pairings. Two *emm* types (*emm25* and *emm81*) were recovered in association with six of the eight FCT-region forms, a finding that further highlights the weakness of defining GAS strains by *emm* type and underscores the promiscuity of GAS genes.

A large fraction of pilin types (68.4%) is also recovered in association with multiple *emm* genotypes (Fig. 6B; Table S8 data). At one extreme is a pilin type (pil053) present in organisms representing 22 different *emm* types. In considering the three major *emm* pattern groupings (A–C, D, and E), 26 pilin types (26.5%) are paired with >1 *emm* pattern grouping, and five pilin types (pil004, pil006, pil023, pil059, and pil065) are each found in association with all three major *emm* pattern groupings (Table S8).

The directionality of HGT for *emm* vs pilin genes is difficult to pinpoint and analyze in a comprehensive way for the entire global strain sample set. SplitsTree graphs of *pilB*34 and *pilB*9 illustrate likely intragenic recombination (Fig 3; Fig S4).

**Fig 7:**
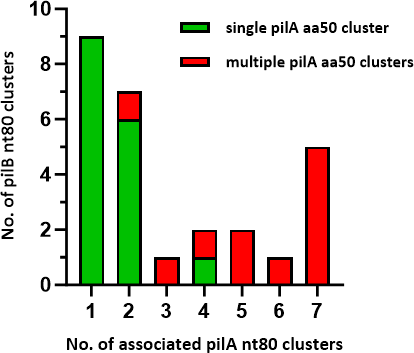
Associations between pilin adhesin and backbone sequence clusters. Bar heights (y-axis) indicate the number of *pilB* nt80 clusters having variable numbers of associated *pilA* nt80 clusters (x-axis). For *pilB* nt80 clusters associated with >1 *pilA* nt80 cluster, the latter are characterized as belonging to a single (green) or multiple (red) PilA aa50 cluster(s).

PilA-PilB compatibility within the pilus heteropolymer may impact the *pilA-pilB* cluster pairings that arise via intra-FCT-region crossover events, as depicted in Fig. 2C. Although genotype does not necessarily equate to phenotype, only 8% of ~21,000 GAS isolates in a meta-analysis could not be T-serotyped (31). T-non-typeability may result from deficiencies in cell surface expression of pilin proteins or lack of coverage by the 21 T-typing reference sera (e.g., Figures 3, S4). Excluding FCT-9 organisms (which lack *pilA*), 67% of *pilB* nt80 clusters co-occur with >1 *pilA* nt80 cluster (Fig. 7; bar height); strikingly, 61% are associated with multiple *pilA* aa50 ancestral-like lineages (red bars). Taken together, the data reveal considerable flexibility in PilA-PilB pairings.

**Fig 8:**
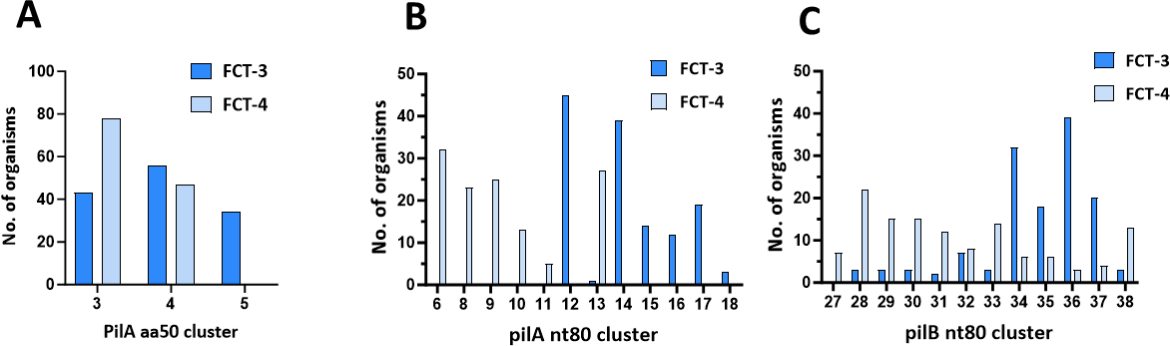
Distribution of pilin sequences across the FCT-3 and FCT-4 regions. For the 258 organisms with unique *emm*-pilin types and FCT-3 or FCT-4 region forms, relative distributions are measured for (**A**) PilA aa50 sequence clusters; (**B**) *pilA* nt80 sequence clusters; and (**C**) *pilB* nt80 sequence clusters. The x-axes designate the cluster number assignments. The *pilA* nt80_16, nt80_17 and nt80_18 clusters (panel B) correspond to the PilA aa50_05 cluster (panel A).

FCT-3 and FCT-4 region forms account for 258 (68.1%) of the 379 unique *emm*-pilin type combinations (Fig. 4A). FCT-34 regions harbor three PilA aa50 ancestral clusters and a single PilB aa50 cluster (Fig. 2A and B). All three PilA aa50 lineages are present in FCT-3, and two are present in FCT-4 (Fig. 8A). FCT-34 regions have 12 *pilA* nt80 clusters and with only one exception, each *pilA*34 nt80 cluster is restricted to either the FCT-3 or FCT-4 region form (Fig. 8B). In contrast, *pilB*34 backbone genes are more versatile, with 43 (of 258 unique *emm*-pilin-type combinations; 16.7%) having *pilB*34 nt80 clusters paired with both FCT-region forms (Fig. 8C). This overall structural design is consistent with the idea that immune escape is facilitated by permissive changes in the backbone pilin, whereas the critical role of PilA in bacterial adherence to human tissue imposes functional constraints.

The PilA34 aa50_5 ancestral cluster is confined to the FCT-3 region among the organisms under study. PilA aa50_5 alleles are distinguished by their relative lack of a coding region for ~229 amino acids and are substantially shorter in length (Table S3). The domain missing in PilA aa50_5, but present in PilA aa50_3 and aa50_4, contains a Cys residue (TCFN consensus/motif) that can form a thioester bond with free amines, a possible mechanism for GAS cell attachment to host tissue (55, 56). The data provide support that PilA products of distinct ancestral lineages may differ in biological function. It should be noted that the PrtF1 gene product of the FCT-4 region (Fig. 1), which differentiates FCT-4 from the FCT-3 region on a gross scale, also has the capacity to act as a “chemical harpoon” via a reactive thioester domain (56).

### Clinico-epidemiological inferences and the global burden of pilin genotypes

There is a rich pool of population-based surveillance for GAS recovered from symptomatic infections at the superficial epithelial layers of the throat or skin, giving rise to pharyngitis or impetigo, respectively. Many such surveys provide a snapshot in time of the prevalence of different *emm* types within a geographically delimited human host population (14). However, few studies to date incorporate genomics, and thereby, pilin genotypes remain largely unknown. By inferring FCT region from the distribution of each *emm* type among the 379 unique *emm*-pilin type combinations (Table S1), the relationships between *emm* and pilin genotypes can be estimated on a global scale.

**Fig 9:**
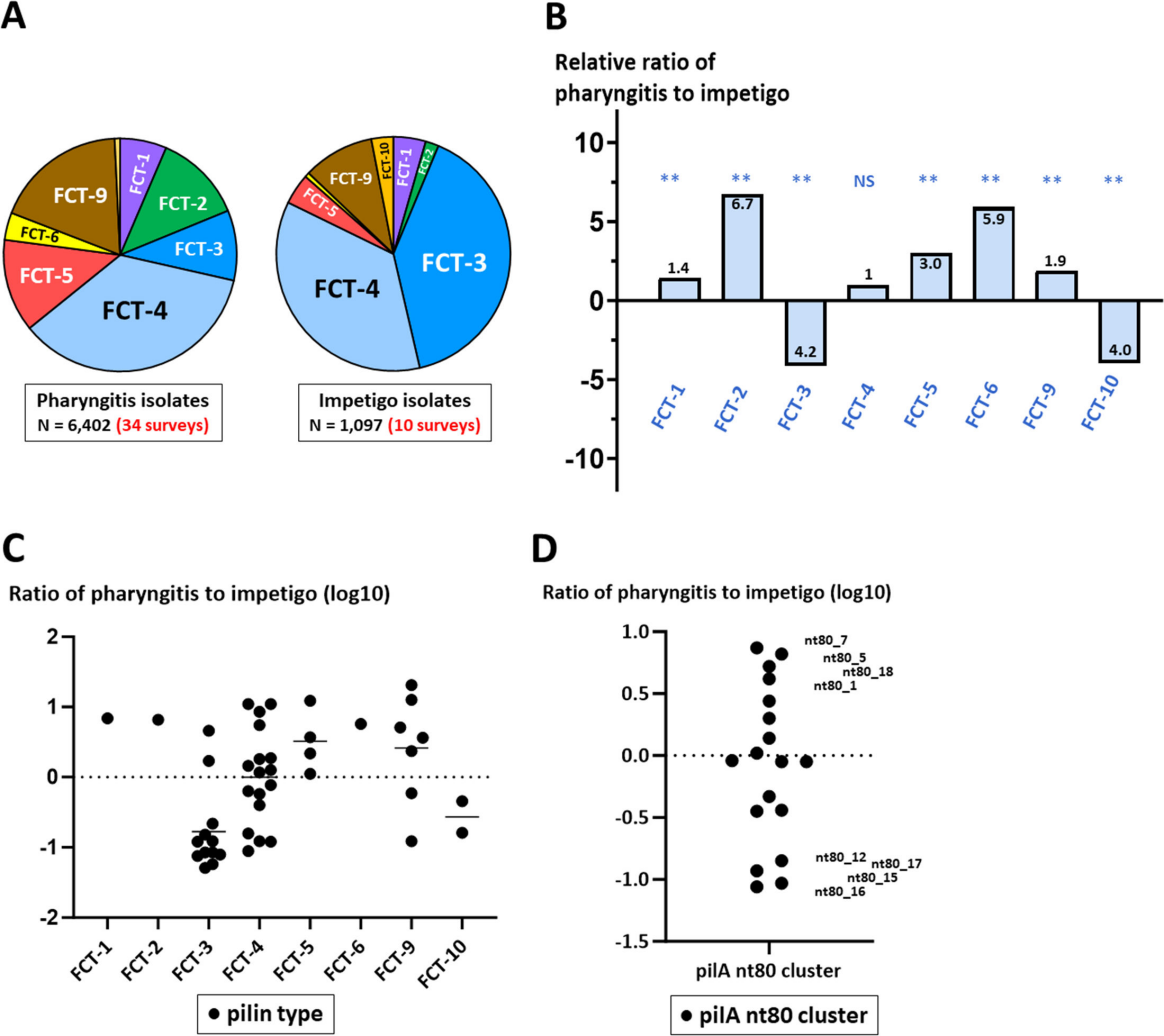
Distribution of inferred pilin genotypes among pharyngitis and impetigo isolates. Data are summarized for pharyngitis (Table S9) and impetigo (Table S10) isolates; excluded are isolates (<1%) with FCT-region forms not determined (n.d.). Inferences for pilin genotypes are based on known *emm* types, using the combined data for Tables S1, S9, and S10. (**A**) Distribution of pharyngitis and impetigo isolates among FCT-region forms for 34 pharyngitis and 10 impetigo surveys. (**B**) Relative ratios of pharyngitis to impetigo isolates, according to FCT-region form; observed vs expected comparisons for pharyngitis vs impetigo isolates, for each FCT-region assignment, were assessed by Fishers exact test and/or χ^2^ with Yates correction (two-tailed); *P* values are highly significant (*P* < 0.01, **) except for FCT-4 (non-significant, NS). (**C and D**) Relative fractional ratios (log_10_) of pharyngitis to impetigo isolates, according to inferred pilin (pil) type (**C**) (raw data are listed in Table S12), and pilA nt80 cluster (**D**); excluded are pilin genotypes comprising <1% of pharyngitis or impetigo isolates; each unique pilin genotype is depicted by ●. For (**C**), mean averages (bars) are shown; for FCT-3 vs FCT-4 pilin types, *t* < 0.01 (unpaired *t*-test and two-tailed).

FCT-region forms are inferred for pharyngitis or impetigo isolates of known *emm* type for 44 population-based surveillance studies (Tables S9 through S11); for *emm* types associated with >1 FCT region form (Fig. 6A), fractional weighting factors are applied. The estimated distribution of FCT-region forms among pharyngitis and impetigo isolates (Fig. 9A) and their relative ratios (Fig. 9B) are calculated. Several FCT regions exhibit strong preferences for pharyngitis: FCT-1, -5, and -9 are paired with diverse sets of *emm* types, whereas 99% of FCT-2 organisms harbor *emm1* and FCT-6 isolates are exclusively *emm2*. In striking contrast, the highly diverse set of FCT-3 isolates display a strong tendency to cause impetigo. Collectively, FCT-4 GAS lack preference for either tissue site of infection, with a ratio of pharyngitis to impetigo near equivalence.

Inferred pilin types were also calculated (Fig. 9C; Table S12). Five pilin types display a >10-fold preference for pharyngitis, whereas seven pilin types show a >10-fold preference for impetigo. Together, the inferred pilA_nt80 clusters have a wide range of affinities for throat or skin infections (Fig. 9D), consistent with the notion that some PilA forms contribute to tissue tropism. The pilin genotypes inferred from population-based collections of GAS provide a sound basis for formulating testable hypotheses on the molecular determinants underlying tissue site preferences for infection.

**Fig 10:**
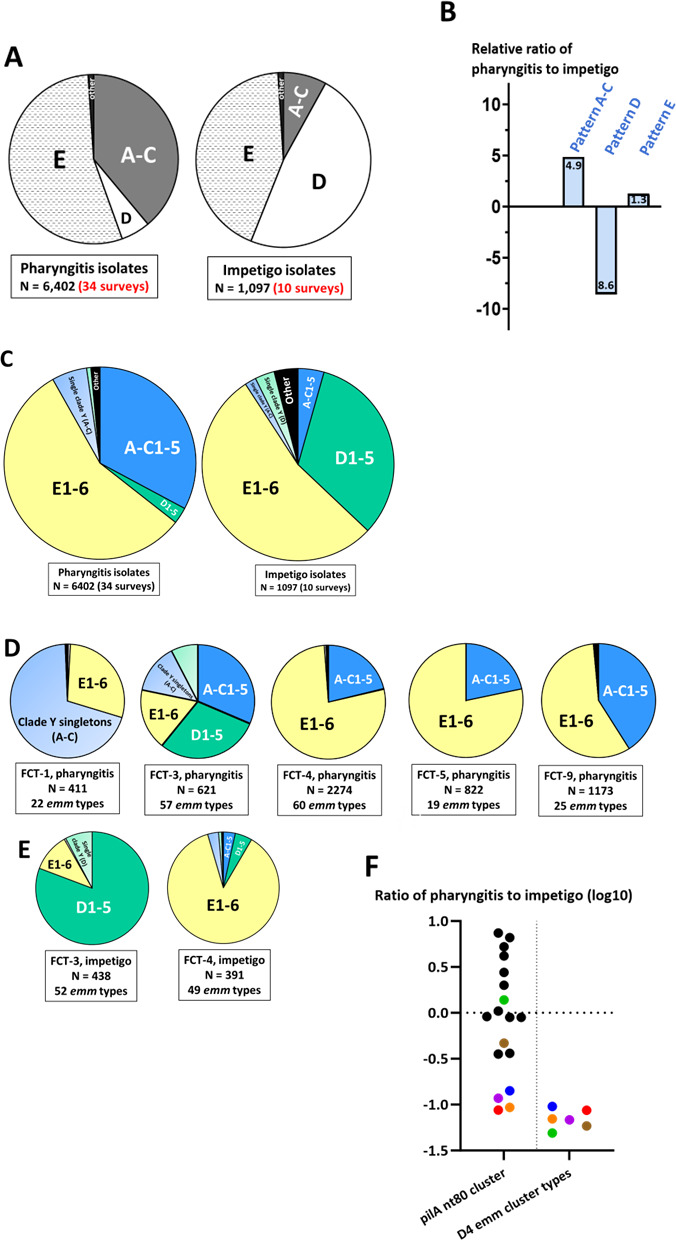
Distribution of inferred *emm* genotypes among pharyngitis and impetigo isolates. Data are summarized for pharyngitis (Table S9) and impetigo (Table S10) isolates; excluded are isolates (<1%) with *emm* pattern groupings that are n.d., rearranged, or mixed for an *emm* type; *emm* cluster is inferred from *emm* type as described (43). (**A**) Distribution of pharyngitis and impetigo isolates among *emm* pattern groupings for 34 pharyngitis and 10 impetigo surveys. (**B**) Relative ratios of pharyngitis to impetigo isolates according to major *emm* pattern grouping. Paired *t* test (two-tailed) for two-way comparisons between the percentage of isolates assigned to *emm* pattern groups A–C vs D, for the 34 collections of pharyngitis isolates (*t* = 7.20E-05) and for the 10 collections of impetigo isolates (*t* = 2.04E-06). (**C**) Distribution of pharyngitis and impetigo isolates among *emm* clusters; “single clade Y” genes represent *emm* genes that occupy single branches in the phylogenetic tree and are subdivided for *emm* patterns A–C and D. (**D and E**) Distribution of pharyngitis (**D**) and impetigo (**E**) isolates among the five main *emm* cluster groups (plus “other”), according to inferred FCT-region form. (**F**) Relative fractional ratios (log_10_) of pharyngitis to impetigo isolates, according to inferred pilA nt80 cluster (left; as shown in Fig. 9D) or a subset of pilA nt80 clusters recovered in association with *emm* cluster D4 (right), which harbors a (putative) plasminogen-binding domain. Colored symbols: red, pilA nt80_16; green, nt80_8; blue, nt80_12; orange, nt80_15; purple, nt80_17; brown, nt80_14.

Despite widespread HGT of *emm* genes, only 6 of the 169 *emm* types (3.6%) were assigned >1 major *emm* pattern grouping (Table S1). Due to strong linkage, *emm* pattern group can be inferred from *emm* type with reasonable accuracy (57, 58) (Tables S9 and S10). The distribution of *emm* pattern among pharyngitis and impetigo isolates (Fig. 10A) and their relative ratios (Fig. 10B) are calculated for the 44 population-based GAS collections. The *emm* pattern A–C isolates display a 4.9-fold preference for pharyngitis over impetigo, whereas *emm* pattern D exhibits the opposite, favoring impetigo over pharyngitis by 8.6-fold (Fig. 10B). Data confirm previous analyses (3, 14, 22, 59) showing that *emm* pattern A–C organisms tend to cause pharyngitis (“throat specialists”), pattern D organisms tend to cause impetigo (“skin specialists”) and pattern E organisms as a group are often recovered from both infected tissue sites (“generalists”). Of note, GAS from asymptomatic carriage or locally invasive skin and soft tissue infections are not considered here. Also, the co-occurrence of pharyngitis and impetigo within a single host population may be limited (60, 61), and *emm* pattern associations with infected tissue may be impacted by geography and/or high prevalence settings.

The widely adopted *emm* “cluster” scheme is based on the “phylogeny” of partial *emm* genes encoding the surface-exposed portion of the M protein fibril (43, 62, 63) and can be deduced from *emm* type (63–67). *emm* cluster excludes the cell wall-spanning SF regions (Fig. S5), and due to poor alignment of *emm* type-specific sequences, it is largely shaped by sequences encoding semi-conserved functional domains that bind human plasma proteins (e.g., IgA, IgG, plasminogen, and fibrinogen) and occupy the central portion of M protein (43, 50, 68). *emm* clusters are highly concordant with *emm* pattern group and adjacent *emm*-like genes (43, 69), further validating the linkage between *emm* type and *emm* pattern and indicative of strong co-selection of non-type-specific portions of *emm* and *emm*-like genes against a backdrop of extensive HGT (Fig. 6).

Linkage of *emm* pattern group to pharyngitis vs impetigo garners additional support from the strong correlations between *emm* cluster and disease, based on extrapolations from the 44 population-based collections of GAS (Fig. 10C). The combinations of *emm* cluster and FCT region reveal genome-wide associations that distinguish subsets pharyngitis and impetigo isolates (Fig. 10D and E). There is a particularly striking association between impetigo and combinations of pilA nt80 cluster and *emm* cluster D4 (encoding a plasminogen-binding domain; Fig. 10F). Data are consistent with a multifactorial basis for tissue site preferences for infection and provide a template for generating hypotheses on pilin structure-function. A goal for pilin genotyping is to facilitate advancements in understanding GAS pathogenesis, as well as vaccine development and new strain emergence.

### Online tools for defining *emm* and pilin gene types

With newly added nucleotide sequences for several (partial) loci, the open-access databases and query tools at https://pubmlst.org/spyogenes (44) can be used to analyze key features of the *emm* and pilin chromosomal regions of GAS. All full-length alleles of *pilA* and *pilB* loci for the 628 GAS genomes (Table S1) are deposited in the online databases. To further facilitate FCT-region assignment, sequences for partial *nra* and *rofA* loci are added (Fig. 1). *emm* types can be ascertained via querying the *emm* typing scheme maintained in the *Streptococcus pyogenes* PubMLST typing database. *emm* pattern assignments are facilitated via a series of queries that include the four SF regions, the upstream *emm* typing primer site, and *mga* lineage (Fig. S5) (13, 70–72).

Instructions for rules-based assignments are detailed in Supplementary Methods. The 379 unique *emm*-pilin type combinations capture a major share of the genetic diversity of this species. However, newly discovered sequences are expected in future work and can be added to the databases upon curation.

## MATERIALS AND METHODS

### Bacteria sampling

Selection of the 628 global and genetically diverse GAS isolates under study was based on available information for *emm* type, MLST, geographic region, and/or year of isolation; isolates with unique genetic features and/or distance in time-space were chosen for in-depth analysis. T-serotyping reference strains were sourced from the CDC. Additional details are provided in Supplementary Methods; validation of sampling for maximal diversity is presented in Table S14.

### Whole-genome sequencing

WGS of 221 new *S. pyogenes* isolates was undertaken using the Illumina platform and *de novo* assembly; additional genomes were previously reported (24) or retrieved through databases [NCTC3000 project (73); GenBank]; all 21 T-typing reference strains originating from CDC stocks were sequenced or re-sequenced. Ten closed (gap-free) WGS were determined by PacBio long-read sequencing, *de novo* assembled using the Hierarchical Genome Assembly Process and Quiver tools, and manually polished and corrected under the Integrative Genomics Viewer (74).

### Phylogenetics and bioinformatics

Cluster analysis using cd-hit (75) was used for pilin loci and applied at varying thresholds in a hierarchical way, wherein clusters were defined at the higher threshold and representatives of that clustering were moved into the next threshold for analysis. The following thresholds were applied: 98%, 95%, 92%, 90%, 88%, 85%, and 80% nt identity, and 70% and 50% amino acid sequence identity. After this step, all alleles are reassigned to the best-fitting cluster at each threshold with cd-hit-2D (aa) and cd-hit-est2D (nt). Sankey diagrams are generated using (76). WGS clustering was performed using PopPUNK (53). Sequence alignments and phylogenetic trees are constructed using MEGA (v.11), Lasergene (v.17), and SplitsTree (v.5) software (77, 78). Pilin and MLST alleles and MLST-based STs are determined and/or assigned using PubMLST with curation permissions (44). *emm* type and *emm* subtype (i.e., partial *emm* allele) are established via databases (*Streptococcus* Laboratory: *S. pyogenes* | CDC) (45) uploaded to PubMLST. *emm* pattern group and FCT-region form are assigned to their respective character states by queries through PubMLST, as detailed in supplementary methods.

### Systematic meta-analysis of pharyngitis and impetigo isolates

The meta-analysis of population-based surveillance studies for GAS is derived from a systematic review (14) and included all collections that satisfied the additional (narrower) inclusion criteria of (i) isolates are clearly defined as recovery from cases of pharyngitis (or tonsillitis) or impetigo; and (ii) >25 isolates could be assigned an *emm* pattern group based on reported *emm* type (21, 22). Adopting the PubMed search criteria applied in reference (14) and including the search terms “pharyngitis” or “impetigo,” there were 333 publications (January 2009 to August 2023), yielding 18 additional population-based samplings of GAS that satisfy the inclusion criteria and report data in a form amenable to our analyses. Pilin-based genotypes of organisms from the population-based surveys were inferred from *emm* type, based on their fractional distribution among the 379 unique *emm*-pilin type combinations reported in Table S1.

### Statistics

Statistical analysis is performed using GraphPad (version 10).

## Data Availability

All alleles and partial alleles listed in Table S1 are available at https://pubmlst.org/spyogenes. Accession numbers for new whole genome sequences are listed in Table S1; related links are as follows: PRJNA1049858, PRJNA395240, PRJNA559889.

## SUPPLEMENTAL MATERIAL

Supplemental material mbio.00693-24-s0001.docx Supplemental Tables and Figures and additional experimental details.

Supplemental tables mbio.00693-24-s0002.xlsx Tables S1, S9, S10, S12, and S13.

ASM does not own the copyrights to Supplemental Material that may be linked to, or accessed through, an article. The authors have granted ASM a non-exclusive, world-wide license to publish the Supplemental Material files. Please contact the corresponding author directly for reuse.
