# Supplementary material for: Recombinational exchange of M-fibril and T-pilus genes generates extensive cell surface diversity in the global group A Streptococcus population
Source: mBio. 2024 Apr 9;15(5):e00693-24. doi: 10.1128/mbio.00693-24 (PMC11078000; doi:10.1128/mbio.00693-24)
Supplement: Supplemental material — Supplemental Tables and Figures and additional experimental details. [file mbio.00693-24-s0001.docx]

**SUPPLEMENT: “**Recombinational Exchange of M-Fibril and T-Pilus Genes Generates Extensive Cell Surface Diversity in the Global Group A Streptococcus Population”, by Bessen *et al.*

*Tables: pages 2-16*

*Figures: pages 17-26*

*Methods: pages 27-29*

*Citations: pages 30-36*

*NOTE: Tables S1, S9, S10, S12 and S13 are uploaded as separate worksheets in an xlsx file.*

**SUPPLEMENTARY TABLES**

**Table S1: Summary genotypes and phenotypes for the 628 GAS isolates.**

Attached xlsx.

Footnotes:

- *pilA*, *pilB*, *pilL* and *fctZ* alleles are deposited at <https://pubmlst.org/spyogenes>
- *emm* pattern and FCT-region forms are established by methods detailed in Supplementary Methods, via sequence queries at <https://pubmlst.org/spyogenes>

# Davies at el. (1), NCTC3000 (2)

^ All 21 T-typing reference strains underwent WGS from CDC stocks; in some instances where genotyping data was confirmed with NCTC3000 strains, the accession number for the latter is listed.

**Table S2: Proposed *pilA*, *pilB* and *pilL* gene nomenclature for GAS pilin genes.**

| FCT-region form | New *pilA* | New *pilB* | New *pilL* | Former adhesin (AP1) | Former backbone (BP) | Former linker  (AP2) |
| --- | --- | --- | --- | --- | --- | --- |
| FCT-1 | *pilA*1 | *pilB*1 | *pilL*1 | *fctX* | *tee* | n/a |
| FCT-2 | *pilA*2 | *pilB*2 | *pilL*2 | *cpa* | SPy0128 | SPy0130 |
| FCT-3 & 4 | *pilA*34 | *pilB*34 | *pilL*34 | *cpa* | *fctA* | *fctB* |
| FCT-5 | *pilA*5 | *pilB*5 | *pilL*5 |  |  |  |
| FCT-10 | *pilA*5 | *pilB*10 | *pilL*10 |  |  |  |
| FCT-9 | n/a | *pilB*9 | *pilL*9 |  |  |  |
| FCT-6 | *pilA*6 | *pilB*6 | *pilL*6 |  |  |  |

**Table S3: Key properties of *pilA* (pilin adhesin) alleles and their products.**

| FCT-region form | Locus name | Start codon | Length (nt) | CWSS ^ |
| --- | --- | --- | --- | --- |
| FCT-1 | *pilA*1 | ATG | 3039-3057 | LPSSG |
| FCT-2 | *pilA*2 | ATG | 2274 | VVPTG |
| FCT-3 & 4 | *pilA*34 | ATG, TTG | 1572-1584 or 2220-2295 | VPPTG, variants (VPSTG, VPPTS) |
| FCT-5 & 10 | *pilA*5 | ATG | 4029-4047 | FPMTG |
| FCT-6 | *pilA*6 | ATG | 2673 | FPKTG |

^ CWSS, cell wall sorting signal.

**Table S4: Key properties of *pilB* (pilin backbone) alleles and their products.**

| FCT-region form | Locus name | Start codon | Length (nt) | Ig-like domain architecture | CWSS ^ |
| --- | --- | --- | --- | --- | --- |
| FCT-1 | *pilB*1 | ATG, GTG | 1650-1659 | 3-Domain | LPSTG |
| FCT-2 | *pilB*2 | ATG | 1023 | 2-Domain | EVPTG |
| FCT-3 & 4 | *pilB*34 | ATG | 1029-1056 | 2-Domain | QVPTG, PIPTG |
| FCT-5 | *pilB*5 | TTG | 2124-2163 | 4-Domain | IPQTG |
| FCT-10 | *pilB*10 | ATG, TTG | 2049 & 2172 | 4-Domain | IPQTG |
| FCT-6 | *pilB*6 | ATG | 1737 | 3-Domain | IPNTG |
| FCT-9 | *pilB*9 | ATG | 1659-1743 or 1935 | 3-Domain | IPNTG |

^ CWSS, cell wall sorting signal.

**Table S5: Cluster assignment for partial *pilB* alleles used in the CDC genomics pipeline** (3).

| CDC *tee* type ^ | Size (bp) | FCT-region | *pilB* locus | *pilB* allele hits (100% id) * | *pilB* nt80 cluster hits (100% id) |
| --- | --- | --- | --- | --- | --- |
| 1 | 60 | FCT-2 | *pilB*2 | 1,2 | 39 |
| 2 | 60 | FCT-6 | *pilB*6 | 1 | 11 |
| 3 | 120 | FCT-34 | *pilB*34 | 10,35,72,76,78,79,80,82,88,89,94,96,123,138 | 33, 35 |
| 4 | 60 | FCT-5 | *pilB*5 | 1,2,4,5,6 | 1 |
| 5 | 120 | FCT-34 | *pilB*34 | 36 | 33 |
| 6 | 60 | FCT-1 | *pilB*1 | 6,12,23 | 25 |
| 8 | 120 | FCT-9 | *pilB*9 | 13,17,23 | 7, 8, 12 |
| 9 | 60 | FCT-34 | *pilB*34 | 17,45,46,47,48,93,95,134, | 32 |
| 11 | 60 | FCT-34 | *pilB*34 | 1,9,12,106 | 31 |
| 12 | 120 | FCT-34 | *pilB*34 | 15,22,40,41,42,43,108,116 | 38 |
| 13 | 120 | FCT-34 | *pilB*34 | 4,20,51,52,53,54,90,101,118,136 | 29, 34 |
| 25 | 60 | FCT-9 | *pilB*9 | 1,2,16,18 | 14 |
| 28 | 60 | FCT-34 | *pilB*34 | 83 | 34 |
| 49 | 120 | FCT-34 | *pilB*34 | 73,75,109 | 36 |
| 58 ^#^ | 120 | n/a | n/a | No hits | n/a |
| 59 | 120 | FCT-34 | *pilB*34 | 6,7,11,13,14,19,30,37,38,39,91,119,129,133 | 28 |
| 81 | 120 | FCT-34 | *pilB*34 | 16,69,70,71,105,112,131 | 31 |
| 83 | 120 | FCT-34 | *pilB*34 | 5,28,29,55,92,100,115,132,139 | 37 |
| 89 | 120 | FCT-34 | *pilB*34 | 8,31,32,33,34,85,110,113,120,125 | 30 |
| 91 | 120 | FCT-34 | *pilB*34 | 2,3,18,21,24,25,26,49,56,57,58,59,99,104,122,126,130 | 34 |
| 92 | 120 | FCT-9 | *pilB*9 | 3,11,15 | 12 |

^ The number designation corresponds with one of the associated *emm* types

* Only 100% id matches (over length of CDC *tee* type) are shown, but a hit is scored as positive in the CDC pipeline if nt sequence identity is >95%

# CDC ‘*tee58*’ matches a distinct alternative FCT-like region that is typical of many FCT-like regions present in SDSE; CDC *tee58* appears to be unique to (some) *emm58* strains.

**Table S6: Cluster assignment for partial *pilB* alleles used in TeeVax** (4, 5).

| TeeVax allele | Size (bp) | FCT-region | *pilB* locus | *pilB* allele hits (100% id) | *pilB* nt80 cluster hits (100% id) |
| --- | --- | --- | --- | --- | --- |
| teevax1_1 | 474 | FCT-34 | *pilB*34 | *pilB*34_84 | 34 |
| teevax1_2 | 375 | FCT-34 | *pilB*34 | *pilB*34_22 | 38 |
| teevax1_3 | 468 | FCT-2 | *pilB*2 | *pilB*2_2 | 39 |
| teevax1_4 | 384 | FCT-34 | *pilB*34 | *pilB*34_107 | 33 |
| teevax1_5 | 474 | FCT-34 | *pilB*34 | *pilB*34_64 | 36 |
| teevax1_6 | 390 | FCT-34 | *pilB*34 | *pilB*34_106 | 27 |
| teevax2_1 | 471 | FCT-34 | *pilB*34 | *pilB*34_140 | 32 |
| teevax2_2 | 378 | FCT-34 | *pilB*34 | *pilB*34_89 | 35 |
| teevax2_3 | 474 | FCT-34 | *pilB*34 | *pilB*34_133 | 28 |
| teevax2_4 | 390 | FCT-34 | *pilB*34 | *pilB*34_33 | 30 |
| teevax2_5 | 471 | FCT-34 | *pilB*34 | *pilB*34_118 | 29 |
| teevax2_6 | 390 | FCT-34 | *pilB*34 | *pilB*34_112 | 31 |
| teevax2_7 | 851 | FCT-34 | *pilB*34 | *pilB*34_55-like ^ | 37-like |
| teevax3_1 | 516 | FCT-1 | *pilB*1 | *pilB*1_6 | 25 |
| teevax3_2 | 543 | FCT-6 | *pilB*6 | *pilB*6_1 | 11 |
| teevax3_3 | 519 | FCT-9 | *pilB*9 | *pilB*9_18 | 14 |
| teevax3_4 | 516 | FCT-1 | *pilB*1 | *pilB*1_17 | 21 |
| teevax3_5 | 561 | FCT-5 | *pilB*5 | *pilB*5_4-like * | 1-like |

^ Seven additional nt at 3’end of teevax_7 do not match *pilB*34_55

* Nine single nt polymorphisms relative to the *pilB*5_4 allele, which is the closest hit

**Table S7: *emm* types recovered in association with multiple pilin types. ^**

| *emm* type | No. of associated pilin types | FCT-1 | FCT-2 | FCT-3 | FCT-4 | FCT-5 | FCT-6 | FCT-9 | FCT-10 |
| --- | --- | --- | --- | --- | --- | --- | --- | --- | --- |
| 3 | 2 |  |  | **pil003** |  | **pil004** |  |  |  |
| 4 | 6 |  |  |  | pil066 | **pil004**  pil0048 |  | **pil008**  pil020  **pil025** |  |
| 8 | 5 |  | **pil001** |  | pil040  pil059  pil114 |  |  | **pil008** |  |
| 9 | 4 |  |  |  | **pil009**  pil045  pil046  pil059 |  |  |  |  |
| 12 | 2 |  |  |  | **pil012** |  |  |  | pil238 |
| 13 | 2 |  |  |  | **pil013** | pil060 |  |  |  |
| 14 | 2 |  |  |  | **pil014**  pil114 |  |  |  |  |
| 15 | 3 | **pil023** |  | pil053 | **pil015** |  |  |  |  |
| 18 | 4 |  |  | **pil018**  pil053  pil074 | **pil015** |  |  |  |  |
| 19 | 3 | **pil023**  pil054 |  |  | **pil009** |  |  |  |  |
| 22 | 5 |  |  |  | pil066  pil077 | pil102 |  | pil020 | **pil022** |
| 24 | 2 | **pil023** |  | pil024 |  |  |  |  |  |
| 25 | 7 | pil110 | **pil001** |  | pil059  pil066 | pil048 |  | **pil025** | pil088 |
| 26 | 2 |  |  |  | **pil028** | **pil004** |  |  |  |
| 28 | 4 |  |  |  | pil021  **pil028**  pil059  pil090 |  |  |  |  |
| 29 | 2 | **pil023** |  | pil070 |  |  |  |  |  |
| 30 | 2 | **pil023** |  | **pil003** |  |  |  |  |  |
| 31 | 2 | **pil023** |  |  |  |  |  | pil055 |  |
| 33 | 2 |  |  | pil033 | pil042 |  |  |  |  |
| 36 | 3 |  |  | pil036  pil053 |  | **pil004** |  |  |  |
| 39 | 2 |  |  |  | **pil009**  pil039 |  |  |  |  |
| 41 | 2 |  |  | pil033  pil043 |  |  |  |  |  |
| 42 | 2 |  |  | **pil003** | pil042 |  |  |  |  |
| 43 | 3 |  |  | pil033  pil043  pil080 |  |  |  |  |  |
| 44 | 2 |  |  |  | **pil011**  pil044 |  |  |  |  |
| 49 | 3 |  |  | pil053 | pil049 |  |  | **pil008**  pil065 |  |
| 50 | 2 |  |  |  | pil066 | **pil004** |  |  |  |
| 52 | 4 |  |  | **pil003**  pil033  pil052  pil064 |  |  |  |  |  |
| 53 | 3 |  |  | pil033  pil053  pil080 |  |  |  |  |  |
| 54 | 2 | pil054 |  | pil070 |  |  |  |  |  |
| 56 | 3 |  |  | pil056  pil083  pil098 |  |  |  |  |  |
| 57 | 3 |  |  | pil043 | **pil009** |  |  | pil057 |  |
| 58 | 2 |  |  |  |  | pil060 |  | pil058 |  |
| 60 | 3 |  |  |  |  | **pil004**  pil060 |  | **pil008** |  |
| 63 | 4 | pil063 |  |  |  | pil232 |  | pil007  pil010 |  |
| 64 |  |  |  | pil053  pil064 |  |  |  |  |  |
| 65 | 3 |  |  | **pil003**  pil072 |  |  |  | pil065 |  |
| 66 | 2 |  |  |  | **pil013**  pil066 |  |  |  |  |
| 68 | 6 | **pil006**  pil068 | **pil001** |  | pil089  pil066  pil106 |  |  |  |  |
| 70 | 3 |  |  | pil053  pil070  pil080 |  |  |  |  |  |
| 71 | 2 |  |  | pil053  pil074 |  |  |  |  |  |
| 73 | 3 |  |  |  | **pil013**  pil044  pil085 |  |  |  |  |
| 75 | 6 | pil110 |  |  |  | **pil004**  pil016  pil060 |  | **pil025**  pil075 |  |
| 76 | 7 | pil076 |  |  | **pil012**  pil039  pil104 |  |  | **pil008**  pil019 | **pil022** |
| 77 | 7 |  |  |  | pil040  pil044  pil059  pil077 | pil102  pil232 |  | pil065 |  |
| 78 | 3 |  |  |  | **pil011**  pil059  pil078 |  |  |  |  |
| 79 | 2 |  |  |  | **pil011** |  |  | pil058 |  |
| 80 | 2 |  |  | pil052  pil080 |  |  |  |  |  |
| 81 | 8 | **pil006**  pil090 |  | pil088 | pil094 | pil048  pil081 |  | pil065 | pil076 |
| 82 | 4 | pil076 |  |  | **pil012**  pil044  pil085 |  |  |  |  |
| 83 | 2 |  |  | pil053  pil083 |  |  |  |  |  |
| 84 | 2 |  |  | pil053 | pil084 |  |  |  |  |
| 85 | 2 |  |  |  | **pil013**  pil085 |  |  |  |  |
| 86 | 3 |  |  | pil043  pil053  pil225 |  |  |  |  |  |
| 89 | 4 |  |  |  | **pil011**  pil089  pil039  pil077 |  |  |  |  |
| 90 | 3 | **pil023** |  |  | **pil013**  pil090 |  |  |  |  |
| 91 | 2 |  |  | pil070  pil083 |  |  |  |  |  |
| 92 | 3 |  |  |  | pil059  pil092 |  |  | pil019 |  |
| 93 | 4 |  |  | pil033  pil052  pil053  pil080 |  |  |  |  |  |
| 95 | 3 | pil095 |  | pil053  pil083 |  |  |  |  |  |
| 97 | 4 |  |  | **pil003**  pil052  pil080  pil098 |  |  |  |  |  |
| 98 | 5 |  |  | pil064  pil080  pil098  pil101  pil225 |  |  |  |  |  |
| 99 | 3 |  |  | pil098  pil099 |  |  |  | pil065 |  |
| 100 | 4 |  |  | pil053  pil056  pil070  pil100 |  |  |  |  |  |
| 101 | 2 |  |  | pil070  pil101 |  |  |  |  |  |
| 102 | 4 | pil115 |  |  | pil059  pil183 | pil102 |  |  |  |
| 103 | 3 |  |  |  | pil059  pil085 |  |  |  | **pil022** |
| 104 | 4 |  |  |  | pil039  pil104  pil106 |  |  |  | **pil022** |
| 105 | 2 |  |  |  | pil085  pil105 |  |  |  |  |
| 106 | 2 |  |  |  | pil059  pil106 |  |  |  |  |
| 108 | 2 | pil095 |  |  | pil070 |  |  |  |  |
| 110 | 4 | pil110 |  |  | pil059  pil077 |  |  |  | pil088 |
| 111 | 2 |  |  | pil070  pil111 |  |  |  |  |  |
| 112 | 4 |  |  |  | pil059  pil090  pil114 |  |  |  |  |
| 113 | 2 |  |  |  | **pil012** |  |  | **pil008** |  |
| 114 | 4 |  |  |  | **pil013**  pil106  pil114 | pil060 |  |  |  |
| 115 | 2 | pil115 |  | pil074 |  |  |  |  |  |
| 116 | 3 |  |  | pil053  pil116  pil225 |  |  |  |  |  |
| 117 | 2 | **pil006** |  | pil117 |  |  |  |  |  |
| 118 | 5 | **pil006** |  | pil074 | **pil013**  pil106 | pil232 |  |  |  |
| 119 | 2 |  |  | pil056  pil098 |  |  |  |  |  |
| 121 | 2 |  |  | pil098 | **pil013** |  |  |  |  |
| 122 | 2 |  |  | **pil005**  pil122 |  |  |  |  |  |
| 123 | 2 |  |  | pil053  pil056 |  |  |  |  |  |
| 124 | 2 |  |  |  | pil039  pil114 |  |  |  |  |
| 165 | 2 |  |  |  |  |  |  | pil075  pil165 |  |
| 166 | 2 |  | **pil001** |  | pil039 |  |  |  |  |
| 168 | 2 |  |  |  | pil039  pil106 |  |  |  |  |
| 169 | 3 |  |  |  | pil066 | pil060 |  | **pil025** |  |
| 177 | 2 |  |  |  | pil177 |  |  | pil019 |  |
| 183 | 5 |  |  |  | **pil013**  pil059  pil077  pil183 |  |  | pil058 |  |
| 186 | 2 |  |  | pil098  pil186 |  |  |  |  |  |
| 192 | 2 |  |  | pil098  pil178 |  |  |  |  |  |
| 207 | 2 |  |  | pil053  pil074 |  |  |  |  |  |
| 209 |  |  |  |  | pil039  pil114 |  |  |  |  |
| 217 | 3 |  |  | pil053  pil217 | pil084 |  |  |  |  |
| 218 | 2 |  |  | pil098 | pil039 |  |  |  |  |
| 223 | 2 |  |  | pil043  pil053 |  |  |  |  |  |
| 225 | 3 |  |  | pil080  pil098  pil225 |  |  |  |  |  |
| 230 | 3 |  |  | pil053  pil080  pil225 |  |  |  |  |  |
| 232 | 2 |  |  |  | pil114 | pil232 |  |  |  |
| 233 | 3 |  |  | pil056 | **pil009** |  |  |  |  |
| 238 | 2 |  |  |  | **pil009** |  |  | pil238 |  |
| 239 | 2 |  |  |  | **pil028** |  |  | pil238 |  |
| stG653 | 2 |  |  |  |  | pil060 |  | pil238 |  |

^ pil types associated with T-serotyping strains are highlighted in **bold**.

**Table S8: Pilin types recovered in association with multiple *emm* types. ^**

| Pilin type | FCT-region form | T-serotype | No. of associated *emm* types | emm pattern  A-C | emm pattern  D | emm pattern  E | REA pattern |
| --- | --- | --- | --- | --- | --- | --- | --- |
| pil001 | FCT-2 | T1 | 7 | 1, 166, 227, 241 |  | 8, 25, 68 |  |
| pil002 | FCT-6 | T2 | 1 ^ |  |  | 2 | null |
| pil003 | FCT-3 | T3 | 9 | 3 | 30, 42, 52, 65, 97, 162, 172, 179 |  |  |
| pil004 | FCT-5 | T4 | 9 | 3, 26, 46 | 36 | 4, 50, 60, 75, 171 |  |
| pil005 | FCT-3 | T5 | 2 | 5 | 122 |  |  |
| pil006 | FCT-1 | T6 | 6 | 6 | 81 | 68,109,117, 118 |  |
| pil008 | FCT-9 | T8 | 6 |  |  | 4, 8, 49, 60, 76, 113 |  |
| pil009 | FCT-4 | T9 | 7 | 19, 39, 57, 233, 238 |  | 9 | stG1750 |
| pil011 | FCT-4 | T11 | 5 |  |  | 11, 44, 78, 79, 89 |  |
| pil012 | FCT-4 | T12 | 4 | 12 |  | 76, 113 | 82 |
| pil013 | FCT-4 | T13 | 10 |  | 85, 121 | 13, 66, 73, 90, 114, 118, 173, 183 |  |
| pil014 | FCT-4 | T14 | 2 | 14, 51 |  |  |  |
| pil015 | FCT-4 |  | 2 |  | 18 | 15 |  |
| pil019 | FCT-9 | T-Imp19 | 3 |  |  | 76, 92, 177 |  |
| pil020 | FCT-9 |  | 2 |  |  | 4, 22 |  |
| pil022 | FCT-10 | T22 | 4 |  |  | 22, 76, 103, 104 |  |
| pil023 | FCT-1 | T23 | 12 | 17, 19, 23, 24, 29, 30, 31, 37, 47 | 32 | 15, 90 |  |
| pil025 | FCT-9 | T25 | 4 |  |  | 4, 25, 75, 169 |  |
| pil027 | FCT-4 | T27 | 1 ^ |  |  | 27 | null |
| pil028 | FCT-4 | T28 | 3 | 26, 239 |  | 28 |  |
| pil089 | FCT-4 | T-B | 2 |  |  | 68, 89 |  |
| pil033 | FCT-3 |  | 8 |  | 33,41, 43, 52, 53,  93 |  | 174, null |
| pil038 | FCT-1 |  | 2 | 38, 193 |  |  |  |
| pil039 | FCT-4 |  | 12 | 39, 137, 218 |  | 76, 89, 104, 124, 166, 168, 180, 209,  231 |  |
| pil040 | FCT-4 |  | 2 |  |  | 8, 77 |  |
| pil042 | FCT-4 |  | 3 |  | 33, 42 |  | 138 |
| pil043 | FCT-3 |  | 6 | 57 | 41, 43, 86, 221, 223 |  |  |
| pil044 | FCT-4 | T44 | 4 |  |  | 44, 73, 77, 82 |  |
| pil048 | FCT-5 |  | 4 |  | 81 | 4, 25, 48 |  |
| pil052 | FCT-3 |  | 4 |  | 52, 80, 93, 97 |  |  |
| pil053 | FCT-3 |  | 22 |  | 18, 36, 53, 70, 71, 83, 86, 93, 95, 100, 116, 123, 147, 207, 217, 223, 230 | 15, 49, 84, 151 |  |
| pil054 | FCT-1 |  | 2 | 19, 54 |  |  |  |
| pil055 | FCT-9 |  | 3 | 31, 55, 229 |  |  |  |
| pil056 | FCT-3 |  | 8 |  | 56, 67, 100, 119, 123, 208, 224, 233 |  |  |
| pil058 | FCT-9 |  | 3 |  |  | 58, 79, 183 |  |
| pil059 | FCT-4 |  | 15 | stG866 | 59 | 8, 9, 25, 28, 77, 78, 92, 102, 103, 106, 110, 112, 183 |  |
| pil060 | FCT-5 |  | 9 | stG653, stG7882 |  | 13, 58, 60, 75, 114, 169, 176 |  |
| pil064 | FCT-3 |  | 5 |  | 52, 64, 98, 120, 185 |  |  |
| pil065 | FCT-9 |  | 6 | 196 | 65, 81, 99 | 49, 77 |  |
| pil066 | FCT-4 |  | 7 |  |  | 4, 22, 25, 50, 66, 68,  169 |  |
| pil070 | FCT-3 |  | 8 | 29 | 54, 70, 91, 100, 101, 108, 111 |  |  |
| pil072 | FCT-3 |  | 2 |  | 65, 72 |  |  |
| pil074 | FCT-3 |  | 7 |  | 18, 71, 74, 115, 118,  191, 207 |  |  |
| pil075 | FCT-9 |  | 2 |  |  | 75, 165 |  |
| pil076 | FCT-1 |  | 3 |  | 81 | 76, 82 |  |
| pil080 | FCT-3 |  | 10 |  | 43, 53, 70, 80, 93, 97, 98, 213, 225, 230 |  |  |
| pil083 | FCT-3 |  | 7 |  | 81, 56, 83, 91, 95, 184, 242 |  |  |
| pil084 | FCT-4 |  | 2 |  | 217 | 84 |  |
| pil085 | FCT-4 |  | 5 |  | 85 | 73, 82, 103 | 105 |
| pil088 | FCT-10 |  | 4 |  | 81 | 25, 88, 110 |  |
| pil090 | FCT-4 |  | 4 |  | 81 | 28, 90, 112 |  |
| pil094 | FCT-3 |  | 3 |  | 81 | 94 | 164 |
| pil095 | FCT-1 |  | 2 | 95, 108 |  |  |  |
| pil098 | FCT-3 |  | 10 |  | 56, 97, 98, 99, 119, 121, 186, 192, 218, 225 |  |  |
| pil099 | FCT-3 |  | 2 |  | 99, 182 |  |  |
| pil101 | FCT-3 |  | 3 |  | 98, 101, 205 |  |  |
| pil102 | FCT-5 |  | 3 |  |  | 22, 77, 102 |  |
| pil104 | FCT-4 |  | 2 |  |  | 76, 104 |  |
| pil106 | FCT-4 |  | 6 |  |  | 68, 104, 106, 114, 118, 168 |  |
| pil110 | FCT-1 |  | 3 |  |  | 25, 75, 110 |  |
| pil114 | FCT-4 |  | 7 | 14 |  | 8, 112, 114, 124, 209, 232 |  |
| pil115 | FCT-1 |  | 3 |  | 115, 216 | 102 |  |
| pil178 | FCT-3 |  | 3 |  | 178, 192 |  | null |
| pil183 | FCT-4 |  | 2 |  |  | 102, 183 |  |
| pil225 | FCT-3 |  | 6 |  | 86, 98, 116, 225, 230 |  | 34 |
| pil232 | FCT-5 |  | 4 |  |  | 63, 77, 118, 232 |  |
| pil238 | FCT-9 |  | 5 | 12, 226, 238, 239, stG653 |  |  |  |

^ One known *emm* type and REA *emm* region lacking an *emm* locus

**Table S9: Meta-analysis of 34 population-based surveys for GAS pharyngitis.**

Table is in attached xlsx.

Footnotes:

^ The number of isolates assigned each FCT-region form is weighted in accordance with data for the 379 unique *emm*-pilin type combinations reported in Table S1.

*Citations for the 34 pharyngitis studies are listed in Table S11.

# *emm* pattern grouping assignments (A-C, D or E) are based on data from this report, references (6, 7), with updated numerical *emm* type nomenclature (3).

**Table S10: Meta-analysis of 10 population-based surveys for GAS impetigo.**

Table is in attached xlsx.

Footnotes

^ The number of isolates assigned each FCT-region form is weighted in accordance with data for the 379 unique *emm*-pilin type combinations reported in Table S1. Up to four *emm* type/FCT-region form combinations reported in impetigo study #10 differ from combinations observed for the 379 unique *emm*-pilin types of this study; data for the four new *emm* type/FCT-region combinations (confirmed or estimated) are incorporated into the weighted factor calculations, shifting categories for 1.16 and 1.44% of pharyngitis and impetigo isolates, respectively.

*Citations for the 10 impetigo studies are listed in Table S11.

# *emm* pattern grouping assignments (A-C, D or E) are based on data from this report, references (4, 5), with updated numerical *emm* type nomenclature (1).

**Table S11: Population-based surveillance studies used in meta-analysis. ***

| Study number | Citation | Infection type | Location | Dates of isolation |
| --- | --- | --- | --- | --- |
| 1 | (8) | pharyngitis | Kuwait | 1980-1989 |
| 2 | (9) | pharyngitis | Ethiopia | 1990 |
| 3 | (10) | pharyngitis | Mexico | 1990 |
| 4 | (11) | pharyngitis | Mexico | 1991-2000 |
| 5 | (12) | pharyngitis | USA | 1993-1994 |
| 6 | (13) | pharyngitis | Italy | 1996-2001 |
| 7 | (14) | pharyngitis | Austria | 1996-2003 |
| 8 | (15) | pharyngitis | Spain | 1996-1999 |
| 9 | (16) | pharyngitis | Germany | 1997 |
| 10 | (17) | pharyngitis | New Zealand | 1997 |
| 11 | (18) | pharyngitis | USA | 1998 |
| 12 | (19) ^ | pharyngitis | USA | 2000-2002 |
| 13 | (20) | pharyngitis | Japan | 2000-2001 |
| 14 | (21) | pharyngitis | India | 2000-2001 |
| 15 | (22) | pharyngitis | Italy | 2000 |
| 16 | (23) | pharyngitis | Italy | 2001-2002 |
| 17 | (24) | pharyngitis | Australia | 2001-2002 |
| 18 | (25) | pharyngitis | USA | 2002-2003 |
| 19 | (26) | pharyngitis | Japan | 2003-2006 |
| 20 | (27) | pharyngitis | India | 2003 |
| 21 | (28) | pharyngitis | Tunisia | 2003 |
| 22 | (29) | pharyngitis | Belgium | 2004 |
| 23 | (29) | pharyngitis | Brazil | 2004 |
| 24 | (30) ^ | pharyngitis | Japan | 2008 |
| 25 | (31) ^ | pharyngitis | India | 2000-2003 |
| 26 | (32) ^ | pharyngitis | India | 2002-2004 |
| 27 | (33) ^ | pharyngitis | Chile | 1996-2007 |
| 28 | (34) ^ | pharyngitis | India | 2009-2011 |
| 29 | (35) ^ | pharyngitis | South Africa | 2008-2011 |
| 30 | (36) ^ | pharyngitis | Portugal | 2002-2008 |
| 31 | (37) ^ | pharyngitis | Mali | 2006-2009 |
| 32 | (38) ^ | pharyngitis | New Zealand (A) | 2015 |
| 33 | (38) ^ | pharyngitis | New Zealand (D) | 2015 |
| 34 | (39) ^ | pharyngitis | Korea | 2017 |
| 1 | (40) ^ | impetigo | Top End, Australia | 1994-1996 |
| 2 | (9) | impetigo | Ethiopia | 1990 |
| 3 | (41) | impetigo | Nepal | 2000 |
| 4 | (29) | impetigo | Brazil | 2004 |
| 5 | (42) | impetigo | Top End, Australia | 2003-2005 |
| 6 | (43) ^ | impetigo | Fiji | 2006 |
| 7 | (32) ^ | impetigo | India | 2002-2004 |
| 8 | (38) ^ | impetigo | New Zealand | 2015 |
| 9 | (44) ^ | impetigo | India | 2007-2009 |
| 10 | (45) ^ | impetigo | The Gambia | 2018 |

*Studies are derived from the original systematic meta-analysis by Steer et al (46), plus added studies from a systematic meta-analysis that extends through August 2023 (^). All studies meet the following criteria: (i), isolates are clearly defined as recovered from cases of pharyngitis (or tonsillitis) or impetigo; (ii), at least 25 isolates could be assigned an *emm* type; (iii), isolates are non-overlapping with other reports; (iv), there is no preselection for additional phenotypes (e.g., antibiotic resistance), and (v), if data are presented in a graphical format (versus raw numbers), the values could be accurately extrapolated. Study numbers are listed in Tables S9 and S10; pharyngitis studies #1-23 and impetigo studies #1-6 were previously analyzed (47).

**Table S12: Fractional ratio of pharyngitis to impetigo isolates based on inferred pilin type.**

Table is in attached xlsx.

Footnotes:

^ Based on the meta-analysis of *emm* type data for pharyngitis isolates (Table S9), the number of isolates assigned each pilin type (i.e., pilA_nt80 and pilB_nt80 cluster combination) is inferred and weighted in accordance with findings for the 379 unique *emm*-pilin type combinations reported in Table S1, and expressed as a % of the total number of pharyngitis isolates analyzed. Only pilin types represented by >1% of either pharyngitis or impetigo isolates are listed.

* As for pharyngitis isolates but based on the meta-analysis data for impetigo isolates (Table S10).

**Table S13: New loci added to PubMLST.**

Table is in attached xlsx.

**Table S14: Validation of the sampling strategy for the 169 unique *emm* types of this study.**

| Collection | No. of unique *emm* types ^ | No. of isolates | No. of *emm* types overlapping with this study (%) | No. of isolates having *emm* types overlapping with this study (%) |
| --- | --- | --- | --- | --- |
| This study | 169 | 628 | n/a | n/a |
| Pharyngitis, 34 surveys # | 130 | 6,402 | 118 (90.8) | 6,375 (99.6) |
| Impetigo, 10 surveys * | 111 | 1,097 | 109 (98.2) | 1,093 (99.6) |

^ Several of the non-overlapping ‘*emm* types’ reported in the meta-analysis surveys are more typical of the *emm*-like downstream gene known as *enn* (Figure S5).

# Based on Table S9 data. Seven of the 12 *emm* types not included in the set of 628 genomes also lack *emm* cluster assignments.

*Based on Table S10 data. One of the two *emm* types not included in the set of 628 genomes also lack *emm* cluster assignments.

**SUPPLEMENTARY FIGURES**

**Figure S1: Schematic of an M-protein fibril and T-protein pilus on the GAS cell surface.** Highly simplified versions of major cell wall-anchored surface proteins, highlighting some key features. The double **~** symbol is meant to signify that the length of the backbone polymer shaft varies widely (i.e., various stages of growth) and also, it can extend to a >1 µm projection. Also note that some pili forms lack adhesin or linker subunits.

**
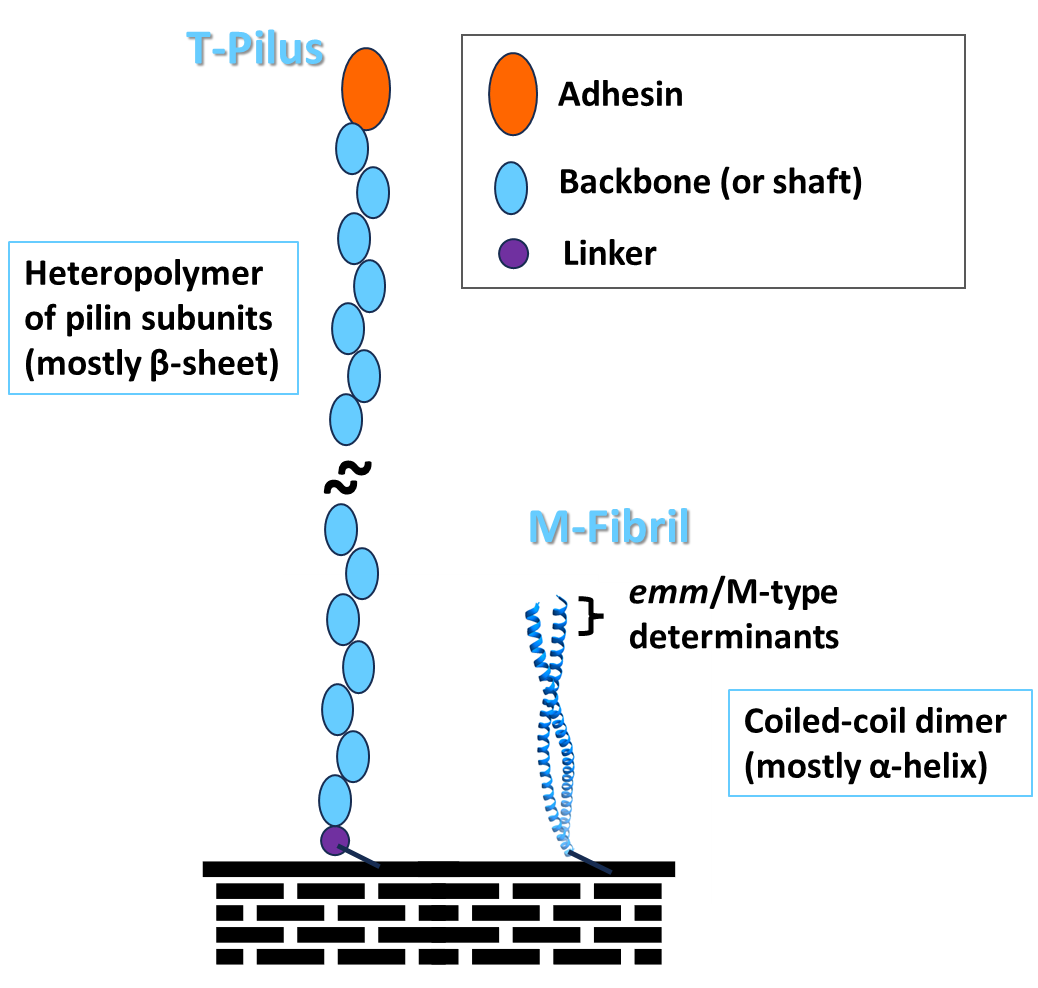
**

**Figure S2: Number of clusters over a range of sequence similarity cutoffs.** (A), *pilA* alleles; and (B), *pilB* alleles. Symbols for loci are *pilA*1, *pilB*1 (▲); *pilA*2, *pilB*2 (♦); *pilA*34, *pilB*34 (●); *pilA*5, *pilB*5 (■); *pilB*9 (▼) and *pilB*10 (□). The number of alleles for each locus is shown in blue. The number of clusters at the nt80 and aa50 thresholds are indicated (red boxes). Cutoff values for % identity for amino acid (aa50, aa70) or nucleotide (nt80, nt85, nt88, nt90, nt92, nt98) sequences, are calculated by cd-hit2 or cd-hit-est2, respectively, with 90% coverage and a word size of 4. Loci *pilA*6 and *pilB*6 each have one allele (data not shown).

**
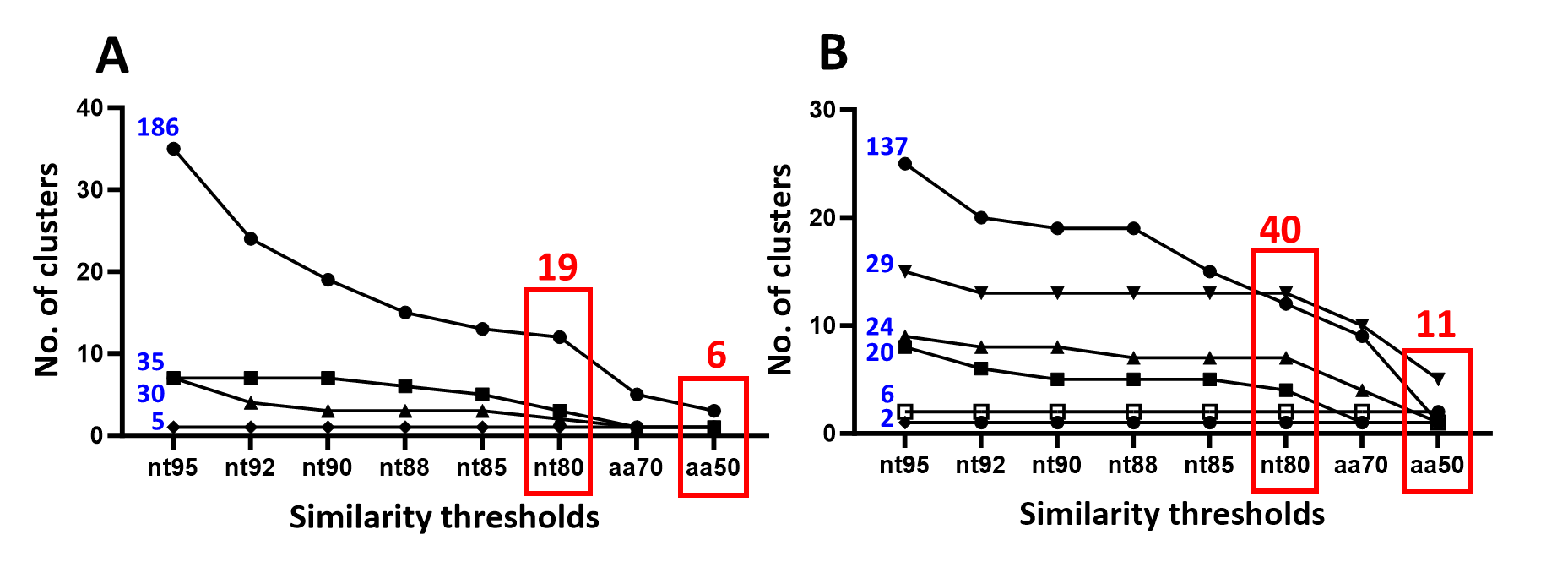
**

**Figure S3: Phylogenetic tree of *pilB*34 alleles and alignment of PilB34 sequences.** Panel (A), Nucleotide sequences for *pilB*34 alleles were aligned using MUSCLE and evolutionary history was inferred using the neighbor-joining method, conducted in MEGA11 using default parameters (48); bootstrapping was performed with 500 replicates and values >90% are shown. The 12 known nt80 clusters (nt80_27 through nt80_38) are indicated at the nodes. Red dots mark three newly discovered *pilB*34 alleles from GAS isolates that are not included in the dataset of 628 GAS genomes used for cd-hit cluster analysis. Panel (B), Clustal Omega alignment of predicted amino acid sequences of full-length *pilB*34 alleles corresponding to the 12 FCT-3 and FCT-4 T-typing reference strains. Uncorrected pairwise distance measures (with global gap removal) for full-length polypeptides indicate % identities ranging from 53% (T28 versus T5, T27 and T44) to 100% (T27 versus T44) (data not shown). The N-terminal end contains a well-conserved signal sequence, and the QVPTG and PIPTG sortase-recognition motifs lie at residue numbers 326 to 330, followed by a highly conserved C-terminal end.

**
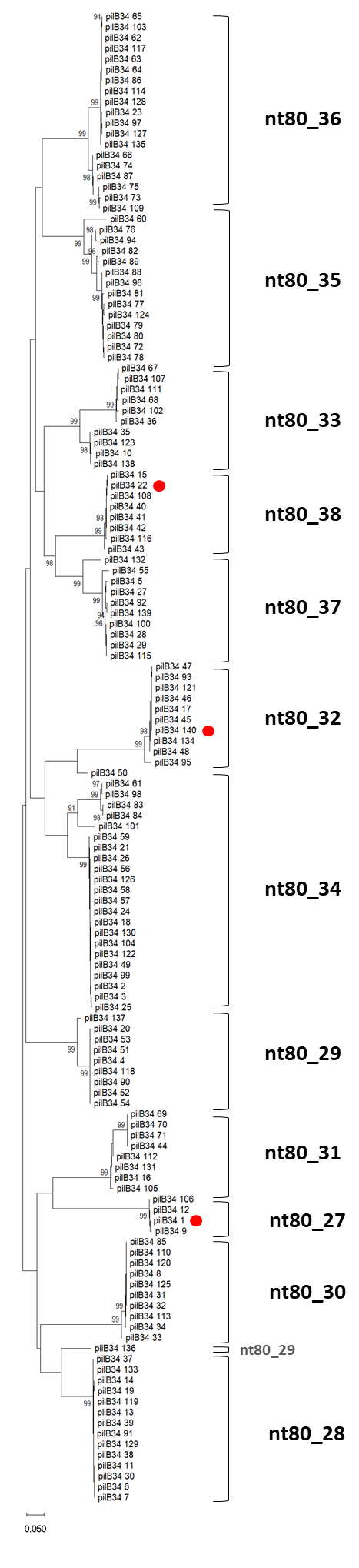
A**

**
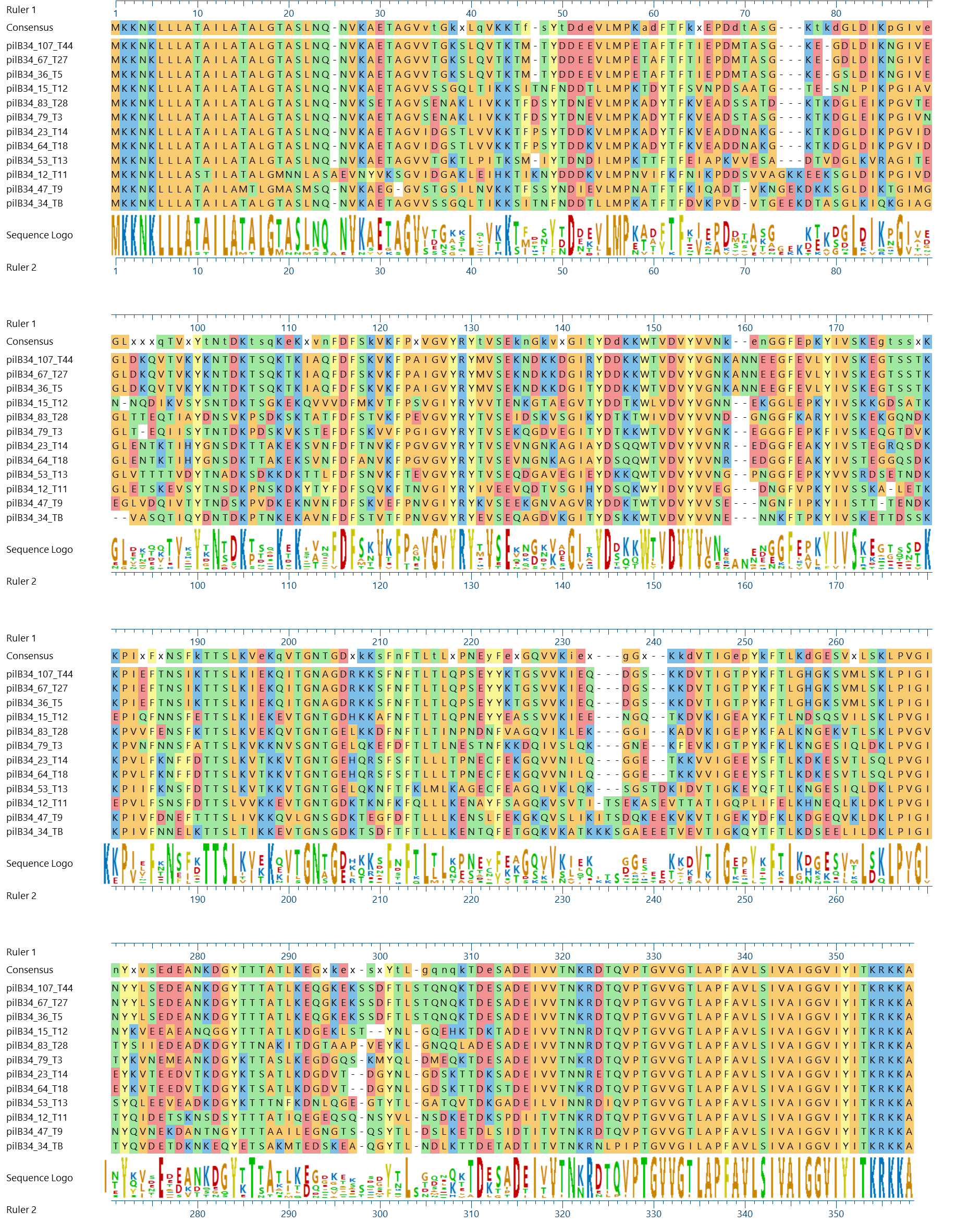
B**

**Figure S4**: **Neighbor-net analysis of *pilB*9 alleles.** Panel (A), The 29 *pilB*9 alleles were aligned by MUSCLE and underwent neighbor-net analysis using SplitsTree v5. Alleles corresponding to the three T-serotype reference strains harboring a *pilB*9 allele are indicated (red font). Also marked are the positions of the 13 nt80 clusters identified by cd-hit (blue). Panel (B), As panel A, with the addition of the *pilB*6 allele (T2 typing strain; orange) and *pilB*-equivalent genes from closely related streptococcal species (green). SDSE, *Streptococcus dysgalactiae* subspecies *equisimilis*; SDSD, *Streptococcus dysgalactiae* subspecies *dysgalactiae*; SD, *Streptococcus dysgalactiae* (non-specified); Scanis, *Streptococcus canis*.

**
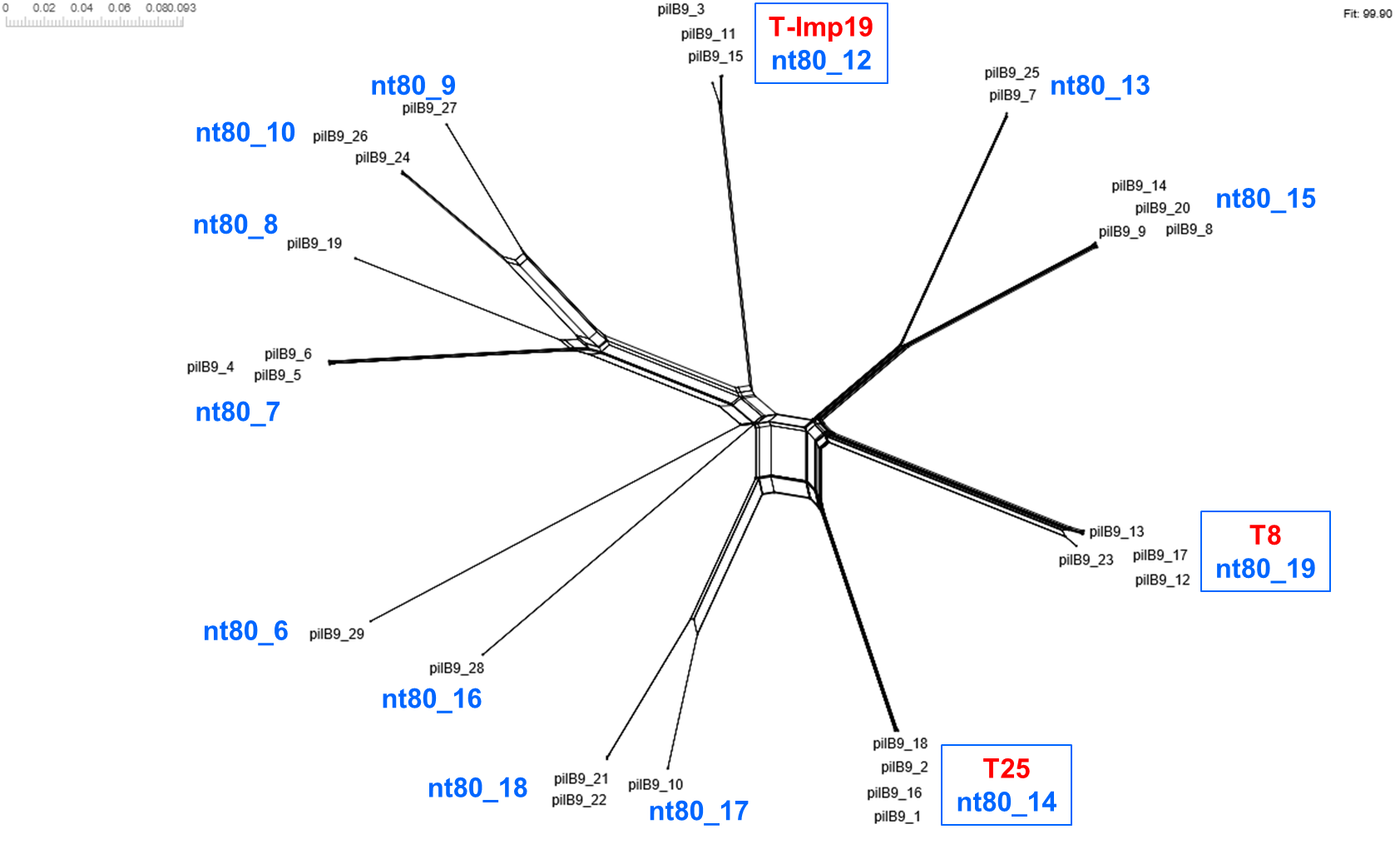
A**

**B**

**
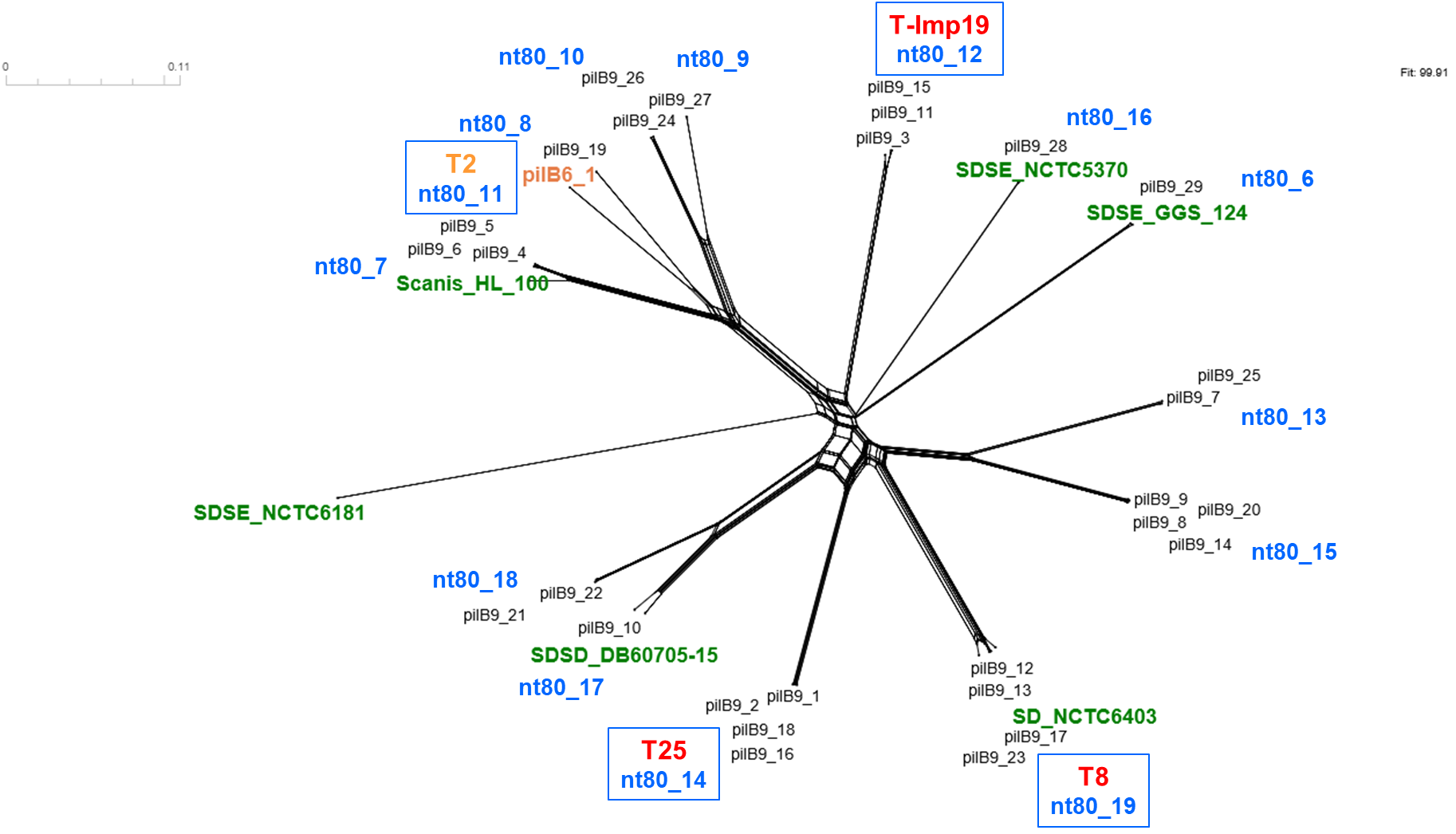
**

**Figure S5: Structure of the *emm* chromosomal region of GAS.** The *emm* chromosomal region harbors the positive transcriptional regulator gene *mga* and one to three *emm* and *emm*-like genes, which are bounded at the 3’ end by the *scpA* gene encoding C5a peptidase. The *emm* gene, defined by its *emm* type determinants near its 5’ end is depicted in red; upstream and downstream flanking *emm*-like genes are often referred to as *mrp* and *enn*, respectively. Patterns A through E are the most common arrangements observed for the *emm* chromosomal region (Table S1); some organisms have rearrangements that likely result from gene duplications or fusions (49) (Table S1; REA). Not shown are transposon genes or the *sic* gene, associated with a small fraction of *emm* types. Panel (A), Highlighted features of the *emm* gene product (top), and the five major *emm* pattern forms, as defined by content and arrangement of subfamily (SF) forms 1 through 4 encoding the cell wall-spanning domain (green) of *emm* and *emm*-like genes (bottom). Panel (B), Arrows point to nt sequences that can be queried via <https://pubmlst.org/spyogenes> to define the *emm* locus and *emm* pattern form: *mga* lineage 1 or 2 (blue); priming site used for *emm* sequence typing (red); SF-specific sequences (green).


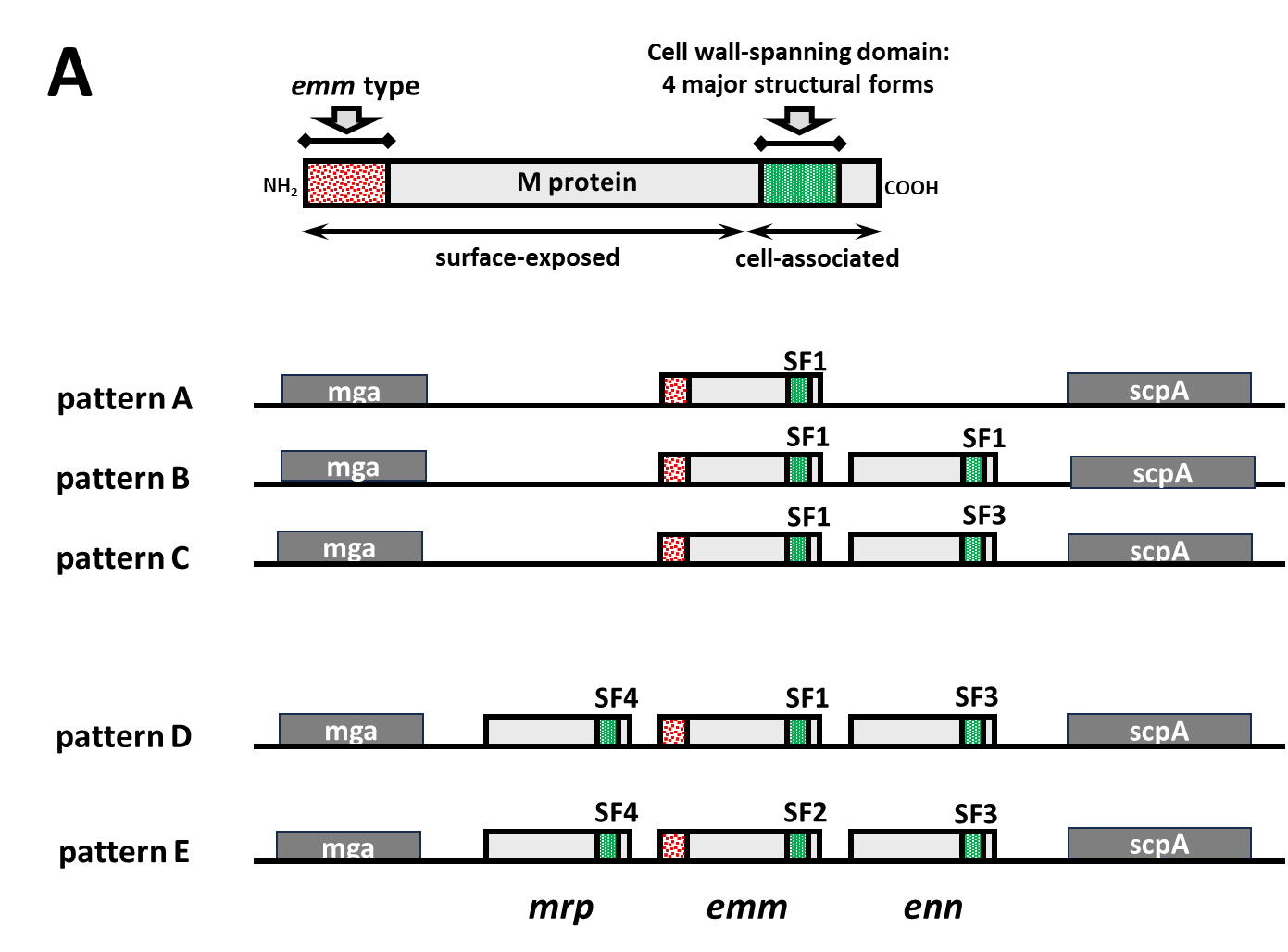


**
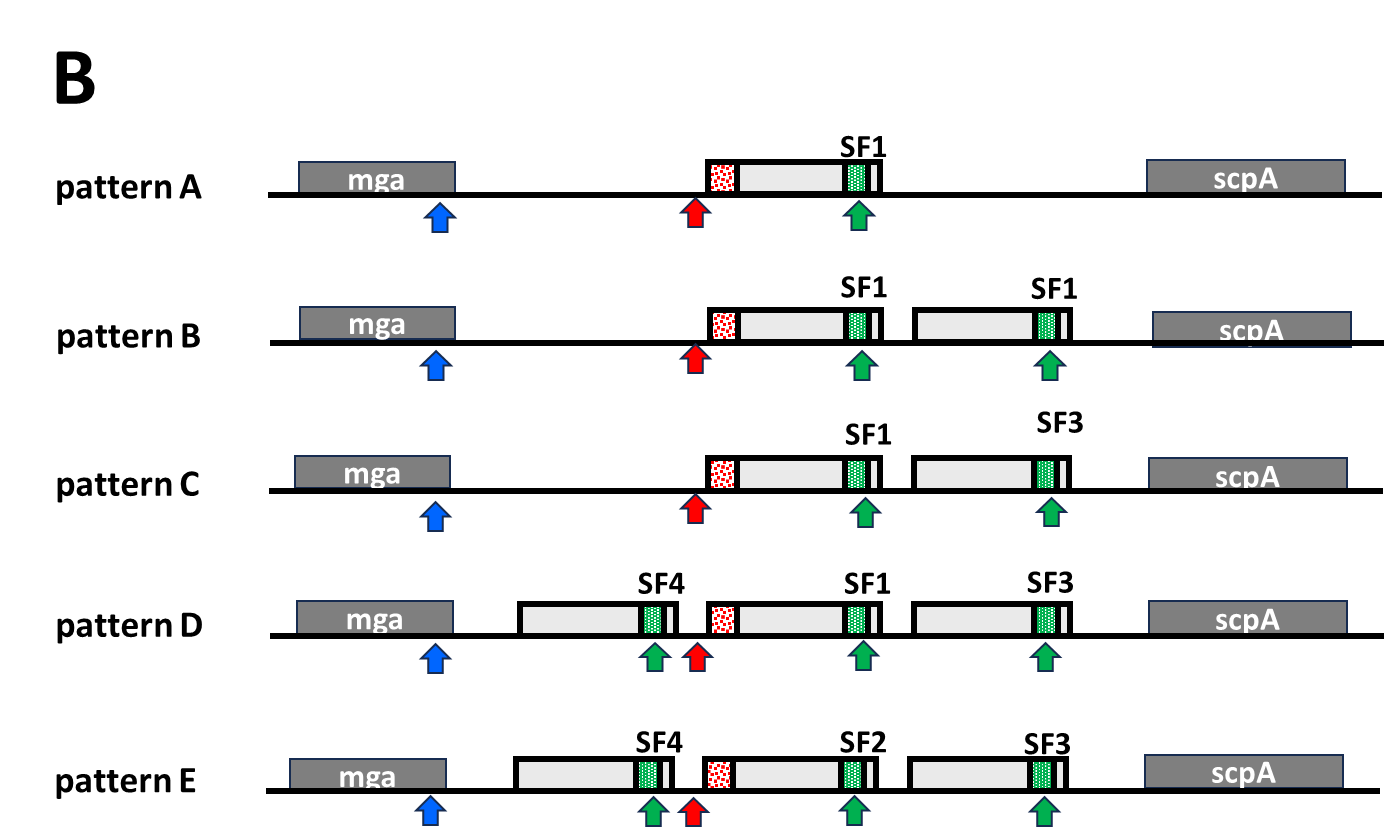
Figure S6: Sample PubMLST queries and *emm* and FCT region analysis.** A genome sequence is queried, and the output Excel file is sorted by ‘start position’ to generate an ordered list of locus hits; the FCT- and *emm*-regions map ~280 kb apart on the ~1.8 Mb genome, equidistant from the origin of replication. Rules-based assignments are made for *emm* (blue) and FCT (green) region forms, in accordance with Figure S5 and Figure 1, respectively; *emm* type is deduced from the *emm* subtype allele assignment. Panels (A), *emm6*, pattern A, FCT-1; (B), *emm53*, pattern D, FCT-3;
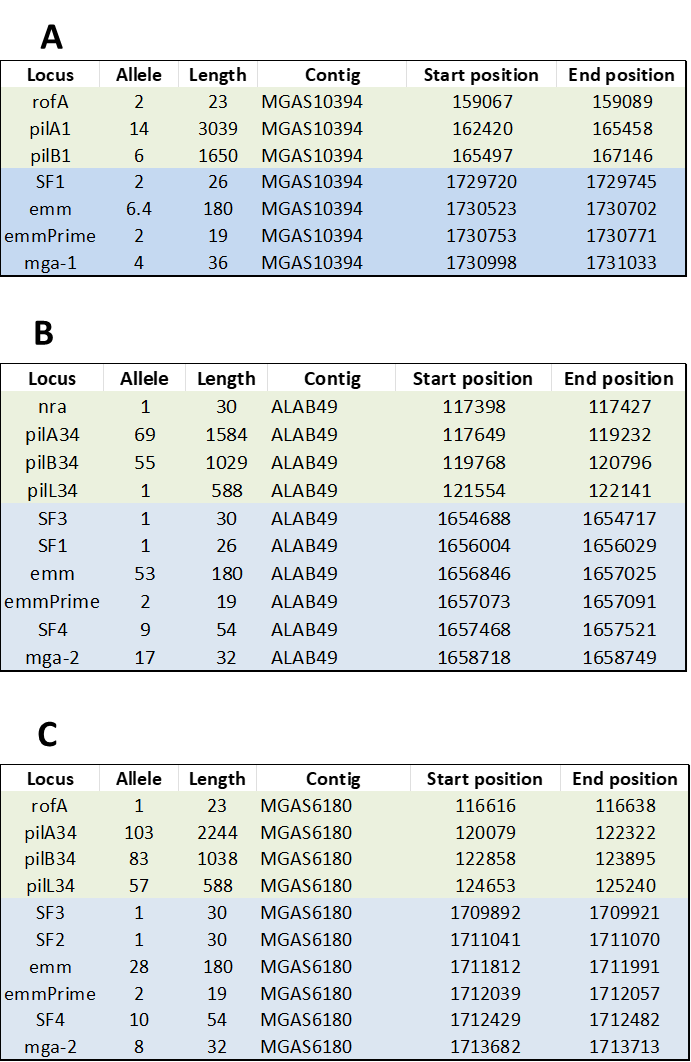
(C), *emm28*, pattern E, FCT-4.

**SUPPLEMENTARY METHODS**

**Bacteria sampling, detailed.** The final set of 628 global and genetically diverse GAS isolates under study was built in several stages over ~10 years. Initially, publicly deposited WGS were selected for diversity of *emm* type and distant ST; the Tables 2 and 3 that were published in (47) provide an approximation of the assembled and draft genomes available for GAS at that time. Initially, ~200 GAS strains from the Bessen lab collection of genetically diverse isolates were chosen for WGS, using similar selection criteria. Thereafter, additional WGS from the Davies collection (N = 1,336) were selected based on unique combinations of *emm* type and distant ST, plus distant geographic region and/or year of isolation (1). In the final stages, WGS having unique *emm* type-distant ST combinations were selected from the CDC collection of iGAS isolates recovered in 2015-2017 (N = 5,565), and all CDC strain stocks of T-typing reference strains underwent WGS. In addition, newly released WGS from the NCTC collection (N = ~80) provided an expanded pool of GAS isolates from the early part of the 20^th^ century (2). It should be noted that several NCTC strains are shared with the CDC collection under different strains names; alternative names are designated in Table S1, and many of the NCTC strains were also sequenced by the CDC or Bessen lab. For some *emm* types lacking MLST diversity (e.g., *emm2*), sampling was expanded in a more concerted attempt to uncover genetic variants. Not all isolates initially selected for genome analysis were chosen for the final set of 628 isolates due to a variety of reasons that included (i), initial errors in *emm* type or MLST assignment; and (ii), lower quality sequence data.

**Data deposits in PubMLST.** Sequence data for portions of the *emm* and FCT chromosomal regions are deposited at <https://pubmlst.org/spyogenes> and new approaches are developed for assigning *emm* type and subtype; *pilA*, *pilB*, *pilL* and *fctZ* alleles; and *emm* pattern and FCT-region forms. New loci added to PubMLST databases are summarized in Table S13.

The current database was built from a genetically global diverse collection of GAS isolates (Table S1) and consequently, the majority of new WGS queries for extant strains are expected to result in positive ‘hits’ and can be readily assigned established *emm* type and pilin alleles; this expectation is evident from our decreasing ability to identify novel alleles (data not shown). The combination of ‘hits’ to numerous other partial alleles allows for quick assignment of *emm* pattern and FCT-region form for most genomes tested; sample data for uncomplicated assignments are depicted in Figure S6.

Missing data/hits may be due to a novel sequence that has not yet been added to the database. If a critical locus is missing, the WGS should be used to query the specific locus in question; new alleles having high homology (>90% identity over 90% length) are identified via the BLASTN-based search function. Rearranged *emm* region sequences split across multiple contigs can be difficult to assess. In instances wherein data analysis is atypical and/or fragmented, confirmation with the annotated genome sequence is essential. For findings consistent with *emm* pattern A, annotations should be checked and/or appropriate loci queried, to help rule in/out patterns B and C.

Assignment of *pilA* and *pilB* nt80 clusters is based on corresponding alleles (Table S1). New *pilA* and *pilB* alleles will be curated and nt80 clusters assigned using phylogenetics wherever possible. Assignment of the pilin type ‘character state’ is based on the combination of *pilA* and *pilB* nt80 clusters (Table S1). Assignment of *emm* type is deduced from the *emm* subtype hit; assignment of *emm* cluster is based on the listing provided in (7). A PDF document that can be downloaded from the https://pubmlst.org/spyogenes website is periodically updated as the databases and character assignments expand. Likewise, new *emm* subtype alleles that are curated by the CDC are uploaded to the PubMLST database (approximately) monthly.

**LITERATURE CITED – SUPPLEMENT ONLY**

1. Davies MR, McIntyre L, Mutreja A, Lacey JA, Lees JA, Towers RJ, Duchene S, Smeesters PR, Frost HR, Price DJ, Holden MTG, David S, Giffard PM, Worthing KA, Seale AC, Berkley JA, Harris SR, Rivera-Hernandez T, Berking O, Cork AJ, Torres R, Lithgow T, Strugnell RA, Bergmann R, Nitsche-Schmitz P, Chhatwal GS, Bentley SD, Fraser JD, Moreland NJ, Carapetis JR, Steer AC, Parkhill J, Saul A, Williamson DA, Currie BJ, Tong SYC, Dougan G, Walker MJ. 2019. Atlas of group A streptococcal vaccine candidates compiled using large-scale comparative genomics. Nat Genet 51:1035-1043.

2. Dicks J, Fazal MA, Oliver K, Grayson NE, Turnbull JD, Bane E, Burnett E, Deheer-Graham A, Holroyd N, Kaushal D, Keane J, Langridge G, Lomax J, McGregor H, Picton S, Quail M, Singh D, Tracey A, Korlach J, Russell JE, Alexander S, Parkhill J. 2023. NCTC3000: a century of bacterial strain collecting leads to a rich genomic data resource. Microb Genom 9.

3. Chochua S, Metcalf BJ, Li Z, Rivers J, Mathis S, Jackson D, Gertz RE, Jr., Srinivasan V, Lynfield R, Van Beneden C, McGee L, Beall B. 2017. Population and Whole Genome Sequence Based Characterization of Invasive Group A Streptococci Recovered in the United States during 2015. MBio 8.

4. Steemson JD, Moreland NJ, Williamson D, Morgan J, Carter PE, Proft T. 2014. Survey of the bp/tee genes from clinical group A streptococcus isolates in New Zealand - implications for vaccine development. J Med Microbiol 63:1670-8.

5. Loh JMS, Rivera-Hernandez T, McGregor R, Khemlani AHJ, Tay ML, Cork AJ, J MR, Moreland NJ, Walker MJ, Proft T. 2021. A multivalent T-antigen-based vaccine for Group A Streptococcus. Sci Rep 11:4353.

6. McMillan DJ, Dreze PA, Vu T, Bessen DE, Guglielmini J, Steer AC, Carapetis JR, Van Melderen L, Sriprakash KS, Smeesters PR. 2013. Updated model of group A Streptococcus M proteins based on a comprehensive worldwide study. Clin Microbiol Infect 19:E222-9.

7. Sanderson-Smith M, De Oliveira DM, Guglielmini J, McMillan DJ, Vu T, Holien JK, Henningham A, Steer AC, Bessen DE, Dale JB, Curtis N, Beall BW, Walker MJ, Parker MW, Carapetis JR, Van Melderen L, Sriprakash KS, Smeesters PR, Group MPS. 2014. A systematic and functional classification of Streptococcus pyogenes that serves as a new tool for molecular typing and vaccine development. J Infect Dis 210:1325-38.

8. Majeed HA, Yousof AM, Rotta J, Havlickpva H, Bahar G, Bahbahani K. 1992. Group A streptococcal strains in Kuwait: a nine-year prospective study of prevalence and associations. Pediatr Infect Dis J 11:295-300; discussion 300-3.

9. Tewodros W, Kronvall G. 2005. M protein gene (emm type) analysis of group a beta-hemolytic streptococci from Ethiopia reveals unique patterns. J Clin Microbiol 43:4369-4376.

10. Mejia LM, Stockbauer KE, Pan X, Cravioto A, Musser JM. 1997. Characterization of group A Streptococcus strains recovered from Mexican children with pharyngitis by automated DNA sequencing of virulence-related genes: unexpectedly large variation in the gene (sic) encoding a complement-inhibiting protein. J Clin Microbiol 35:3220-4.

11. Espinosa LE, Li ZY, Barreto DG, Jaimes EC, Rodriguez RS, Sakota V, Facklam RR, Beall B. 2003. M protein gene type distribution among group A streptococcal clinical isolates recovered in Mexico City, Mexico, from 1991 to 2000, and Durango, Mexico, from 1998 to 1999: Overlap with type distribution within the United States. J Clin Microbiol 41:373-378.

12. Kiska DL, Thiede B, Caracciolo J, Jordan M, Johnson D, Kaplan EL, Gruninger RP, Lohr JA, Gilligan PH, Denny FW, Jr. 1997. Invasive group A streptococcal infections in North Carolina: epidemiology, clinical features, and genetic and serotype analysis of causative organisms. J Infect Dis 176:992-1000.

13. Creti R, Cardona F, Pataracchia M, Hunolstein CV, Cundari G, Romano A, Orefici G. 2004. Characterisation of group A streptococcal (GAS) isolates from children with tic disorders. Indian J Med Res 119 Suppl:174-8.

14. Eisner A, Leitner E, Feierl G, Kessler HH, Marth E. 2006. Prevalence of emm types and antibiotic resistance of group A streptococci in Austria. Diagn Microbiol Infect Dis 55:347-50.

15. Alberti S, Garcia-Rey C, Dominguez MA, Aguilar L, Cercenado E, Gobernado M, Garcia-Perea A. 2003. Survey of emm gene sequences from pharyngeal Streptococcus pyogenes isolates collected in Spain and their relationship with erythromycin susceptibility. J Clin Microbiol 41:2385-2390.

16. Brandt CM, Spellerberg B, Honscha M, Truong ND, Hoevener B, Lutticken R. 2001. Typing of Streptococcus pyogenes strains isolated from throat infections in the region of Aachen, Germany. Infection 29:163-165.

17. Dierksen KP, Inglis M, Tagg JR. 2000. High pharyngeal carriage rates of Streptococcus pyogenes in Dunedin school children with a low incidence of rheumatic fever. N Z Med J 113:496-9.

18. Haukness HA, Tanz RR, Thomson RB, Jr., Pierry DK, Kaplan EL, Beall B, Johnson D, Hoe NP, Musser JM, Shulman ST. 2002. The heterogeneity of endemic community pediatric group a streptococcal pharyngeal isolates and their relationship to invasive isolates. J Infect Dis 185:915-20.

19. Shulman ST, Tanz RR, Kabat W, Kabat K, Cederlund E, Patel D, Li Z, Sakota V, Dale JB, Beall B. 2004. Group A streptococcal pharyngitis serotype surveillance in North America, 2000-2002. Clin Infect Dis 39:325-32.

20. Ma X, Kikuta H, Ishiguro N, Yoshioka M, Ebihara T, Murai T, Kobayashi I, Kobayashi K. 2002. Association of the prtF1 gene (encoding fibronectin-binding protein F1) and the sic gene (encoding the streptococcal inhibitor of complement) with emm types of group A streptococci isolated from Japanese children with pharyngitis. J Clin Microbiol 40:3835-7.

21. Dey N, McMillan DJ, Yarwood PJ, Joshi RM, Kumar R, Good MF, Sriprakash KS, Vohra H. 2005. High diversity of group A Streptococcal emm types in an Indian community: the need to tailor multivalent vaccines. Clin Infect Dis 40:46-51.

22. Dicuonzo G, Gherardi G, Lorino G, Angeletti S, DeCesaris M, Fiscarelli E, Bessen DE, Beall B. 2001. Group A streptococcal genotypes from pediatric throat isolates in Rome, Italy. J Clin Microbiol 39:1687-1690.

23. Lorino G, Gherardi G, Angeletti S, De Cesaris M, Graziano N, Maringhini S, Merlino F, Di Bernardo F, Dicuonzo G. 2006. Molecular characterisation and clonal analysis of group A streptococci causing pharyngitis among paediatric patients in Palermo, Italy. Clin Microbiol Infect 12:189-92.

24. Rogers S, Commons R, Danchin MH, Selvaraj G, Kelpie L, Curtis N, Robins-Browne R, Carapetis JR. 2007. Strain prevalence, rather than innate virulence potential, is the major factor responsible for an increase in serious group A streptococcus infections. J Infect Dis 195:1625-33.

25. Richter SS, Heilmann KP, Beekmann SE, Miller NJ, Miller AL, Rice CL, Doern CD, Reid SD, Doern GV. 2005. Macrolide-resistant Streptococcus pyogenes in the United States, 2002-2003. Clin Infect Dis 41:599-608.

26. Wajima T, Murayama SY, Sunaoshi K, Nakayama E, Sunakawa K, Ubukata K. 2008. Distribution of emm type and antibiotic susceptibility of group A streptococci causing invasive and noninvasive disease. J Med Microbiol 57:1383-8.

27. Sagar V, Bakshi DK, Nandi S, Ganguly NK, Kumar R, Chakraborti A. 2004. Molecular heterogeneity among north Indian isolates of Group A Streptococcus. Lett Appl Microbiol 39:84-8.

28. Mzoughi R, Bouallegue O, Selmi H, Ben Said H, Essoussi AS, Jeddi M. 2004. Group A streptococci in children with acute pharyngitis in Sousse, Tunisia. East Mediterr Health J 10:488-93.

29. Smeesters PR, Vergison A, Campos D, de Aguiar E, Deyi VY, Van Melderen L. 2006. Differences between Belgian and Brazilian Group A Streptococcus Epidemiologic Landscape. PLoS ONE 1:e10.

30. Okabe T, Takeda S, Hida M, Narisada T. 2011. Study of T serotypes and Emm genotypes of Streptococcus pyogenes in children with pharyngitis and tonsillitis. J Nippon Med Sch 78:174-7.

31. Dhanda V, Vohra H, Kumar R. 2011. Virulence potential of Group A streptococci isolated from throat cultures of children from north India. Indian J Med Res 133:674-80.

32. Kumar R, Chakraborti A, Aggarwal AK, Vohra H, Sagar V, Dhanda V, Sharma YP, Majumdar S, Hoe N, Krause RM. 2012. Streptococcus pyogenes pharyngitis & impetigo in a rural area of Panchkula district in Haryana, India. Indian J Med Res 135:133-6.

33. Wozniak A, Rojas P, Rodriguez C, Undabarrena A, Garrate C, Riedel I, Roman JC, Kalergis AM, Garcia P. 2012. M-protein gene-type distribution and hyaluronic acid capsule in group A Streptococcus clinical isolates in Chile: association of emm gene markers with csrR alleles. Epidemiol Infect 140:1286-1295.

34. Balaji K, Thenmozhi R, Prajna L, Dhananjeyan G, Pandian SK. 2013. Comparative analysis of emm types, superantigen gene profiles and antibiotic resistance genes among Streptococcus pyogenes isolates from ocular infections, pharyngitis and asymptomatic children in south India. Infect Genet Evol 19:105-12.

35. Engel ME, Muhamed B, Whitelaw AC, Musvosvi M, Mayosi BM, Dale JB. 2014. Group A streptococcal emm type prevalence among symptomatic children in Cape Town and potential vaccine coverage. Pediatr Infect Dis J 33:208-10.

36. Silva-Costa C, Carrico JA, Ramirez M, Melo-Cristino J. 2014. Scarlet fever is caused by a limited number of Streptococcus pyogenes lineages and is associated with the exotoxin genes ssa, speA and speC. Pediatr Infect Dis J 33:306-10.

37. Tapia MD, Sow SO, Tamboura B, Keita MM, Berthe A, Samake M, Nataro JP, Onwuchekwa UO, Penfound TA, Blackwelder W, Dale JB, Kotloff KL. 2015. Streptococcal pharyngitis in schoolchildren in Bamako, Mali. Pediatr Infect Dis J 34:463-8.

38. Williamson DA, Smeesters PR, Steer AC, Morgan J, Davies M, Carter P, Upton A, Tong SY, Fraser J, Moreland NJ. 2016. Comparative M-protein analysis of Streptococcus pyogenes from pharyngitis and skin infections in New Zealand: Implications for vaccine development. BMC Infect Dis 16:561.

39. Kim S, Lee S, Park H, Kim S. 2019. Predominance of emm4 and antibiotic resistance of Streptococcus pyogenes in acute pharyngitis in a southern region of Korea. J Med Microbiol 68:1053-1058.

40. Bessen DE, Carapetis JR, Beall B, Katz R, Hibble M, Currie BJ, Collingridge T, Izzo MW, Scaramuzzino DA, Sriprakash KS. 2000. Contrasting molecular epidemiology of group A streptococci causing tropical and non-tropical infections of the skin and throat. J Infect Dis 182:1109-1116.

41. Sakota V, Fry AM, Lietman TM, Facklam RR, Li ZY, Beall B. 2006. Genetically diverse group A streptococci from children in Far-Western Nepal share high genetic relatedness with isolates from other countries. J Clin Microbiol 44:2160-2166.

42. McDonald MI, Towers RJ, Fagan P, Carapetis JR, Currie BJ. 2007. Molecular typing of Streptococcus pyogenes from remote Aboriginal communities where rheumatic fever is common and pyoderma is the predominant streptococcal infection. Epidemiol Infect 135:1398-405.

43. Steer AC, Magor G, Jenney AW, Kado J, Good MF, McMillan D, Batzloff M, Carapetis JR. 2009. emm and C-repeat region molecular typing of beta-hemolytic Streptococci in a tropical country: implications for vaccine development. J Clin Microbiol 47:2502-9.

44. Chaudhary P, Kumar R, Sagar V, Sarkar S, Singh R, Ghosh S, Singh S, Chakraborti A. 2018. Assessment of Cpa, Scl1 and Scl2 in clinical group A streptococcus isolates and patients from north India: an evaluation of the host pathogen interaction. Res Microbiol 169:11-19.

45. Bah SY, Keeley AJ, Armitage EP, Khalid H, Chaudhuri RR, Senghore E, Manneh J, Tilley L, Marks M, Darboe S, Sesay AK, de Silva TI, Turner CE, Group MSAS. 2023. Genomic Characterization of Skin and Soft Tissue Streptococcus pyogenes Isolates from a Low-Income and a High-Income Setting. mSphere 8:e0046922.

46. Steer AC, Law I, Matatolu L, Beall BW, Carapetis JR. 2009. Global emm type distribution of group A streptococci: systematic review and implications for vaccine development. Lancet Infect Dis 9:611-6.

47. Bessen DE, McShan WM, Nguyen SV, Shetty A, Agrawal S, Tettelin H. 2015. Molecular epidemiology and genomics of group A Streptococcus. Infect Genet Evol 33:393-418.

48. Tamura K, Stecher G, Kumar S. 2021. MEGA11: Molecular Evolutionary Genetics Analysis Version 11. Mol Biol Evol 38:3022-3027.

49. Frost HR, Davies MR, Delforge V, Lakhloufi D, Sanderson-Smith M, Srinivasan V, Steer AC, Walker MJ, Beall B, Botteaux A, Smeesters PR. 2020. Analysis of Global Collection of Group A Streptococcus Genomes Reveals that the Majority Encode a Trio of M and M-Like Proteins. mSphere 5.
